# Network analysis reveals common host protein/s modulating pathogenesis of neurotropic viruses

**DOI:** 10.1038/srep32593

**Published:** 2016-09-01

**Authors:** Sourish Ghosh, Sriparna Mukherjee, Nabonita Sengupta, Arunava Roy, Dhritiman Dey, Surajit Chakraborty, Dhrubajyoti Chattopadhyay, Arpan Banerjee, Anirban Basu

**Affiliations:** 1 National Brain Research Centre, Manesar, Haryana-122051, India; 2 Department of Biochemistry, University of Calcutta, Kolkata- 700019, India; 3 Amity University Kolkata, Newtown, Kolkata-700135, India.

## Abstract

Network analysis through graph theory provides a quantitative approach to characterize specific proteins and their constituent assemblies that underlie host-pathogen interactions. In the present study, graph theory was used to analyze the interactome designed out of 50 differentially expressing proteins from proteomic analysis of Chandipura Virus (CHPV, Family: Rhabdoviridae) infected mouse brain tissue to identify the primary candidates for intervention. Using the measure of degree centrality, that quantifies the connectedness of a single protein within a milieu of several other interacting proteins, DJ-1 was selected for further molecular validation. To elucidate the generality of DJ-1’s role in propagating infection its role was also monitored in another RNA virus, Japanese Encephalitis Virus (JEV, Family: Flaviviridae) infection. Concurrently, DJ-1 got over-expressed in response to reactive oxygen species (ROS) generation following viral infection which in the early phase of infection migrated to mitochondria to remove dysfunctional mitochondria through the process of mitophagy. DJ-1 was also observed to modulate the viral replication and interferon responses along with low-density lipoprotein (LDL) receptor expression in neurons. Collectively these evidences reveal a comprehensive role for DJ-1 in neurotropic virus infection in the brain.

Identification of key proteins in a host proteome is crucial for targeted therapeutic strategy for neurotropic virus infections. Japanese Encephalitis Virus (JEV) and Chandipura Virus (CHPV) (both RNA viruses) have been ranked among the potential agents that accounted for over 6000 deaths in the last 6 years in India due to Acute Encephalitis Syndrome (AES) (The Indian Express, Mar, 2015). Several previous approaches attempted at targeting specific viral proteins involved in CHPV and JEV inflammation or randomly tested drug targets to impede the progression of encephalitis disease^1^,^2^,^3^. Due to this high mutation rate, previous attempts to develop anti-viral therapies targeting viral proteins or genes against RNA viruses turned out to be ineffective^4^,^5^,^6^,^7^. Since these viruses rapidly multiply within their hosts and lead to encephalitis within 2−3 days, often the process of identification and then deciding on the anti-viral therapy for that particular virus exacerbates the situation. In addition, often multiple RNA viruses infect the same host^8^,^9^,^10^. Thus, modern approaches in developing anti-viral therapies rely on understanding the host system which the RNA viruses manoeuvre to facilitate their survival^11^,^12^. Since most of these rapidly multiplying RNA viruses do not integrate themselves with the host genome and prefer to utilize the host proteins, it is reasonable to analyze the host proteome to “fish out” those “common” proteins and pathways on which these viruses depend in order to frame a common therapeutic strategy against these viruses.

Traditional protein-protein interaction (PPI) studies related to host-viral interactions attempted at studying these interactions in isolation^13^,^14^. Studying PPI in isolation i.e. interaction of individual proteins with a particular viral protein, ignores the global effect of the virus in the host. Systems biology approaches have been applied to reveal systematic trends in host-pathogen interaction networks, e.g., viruses tend to target host protein interaction hubs^15^. Although this approach helps in analyzing the role of a group of host proteins in a viral pathogenesis, it nonetheless fails to screen the important protein/s that should be therapeutically targeted. Prioritization algorithms have been applied of late to find an answer to this problem^16^. One can take measures of graph theory to address this issue, e.g., degree centrality assigns a score to each node based on their connectivity within the network. The node with the highest degree centrality score is considered to be the most important node within that network. Moreover it has been observed that the node with highest degree centrality in a complex network holds its strategic position even if the network is expanded^17^.

In our present approach we tried to “fish out” the strategic host protein in the infection pathway and validate its role in neuropathogenesis. We specifically targeted CHPV and JEV in order to propose a common therapeutic strategy. Primarily we identified a set of host proteins that get differentially modulated by CHPV infection in an established mouse infection model. The identified proteins were mapped into a PPI network to develop an interactome. Applying parameters of graph theory such as degree centrality to the derived interactome, we were able to identify the importance of DJ-1 upregulation in mediating infection.

Protein deglycase or DJ-1 has been implicated to have a neuroprotective role in sporadic cases of Parkinson’s disease and other neurodegenerative disease^18^. Reduced mitochondrial complex-1 activity accompanied by increased oxidative stress has been reported to recruit DJ-1 to mitochondria following either depolarization of the mitochondrial membrane or increased cellular oxidative stress^19^. DJ-1 has been reported to play critical role in various other processes like transcriptional regulation, chaperone activities, apart from its regulatory role in ROS generation^20^. One of the important transcriptional regulatory roles of DJ-1 has been in co-activating low-density lipoprotein receptor (LDLR) transcription along with Sterol Regulatory Element Binding Protein-2 (SREBP-2)^21^. Triggering of oxidative stress, mitochondrial dysfunction and mitophagy following viral infections are common events. In our present venture we tried to explore the role of DJ-1 in case of neurotropic virus infection in neurons. Our investigation revealed the upregulation of DJ-1 during neurodegeneration following CHPV and JEV infection. Also as previously reported LDLR play important role in CHPV infection^22^, we were inquisitive to know that whether DJ-1 has a regulatory effect on LDLR in neurons and hence modulates the life cycle of both CHPV and JEV.

## Results

### Bioinformatic analysis of protein networks to identify key host protein/s

**Figure 1:**
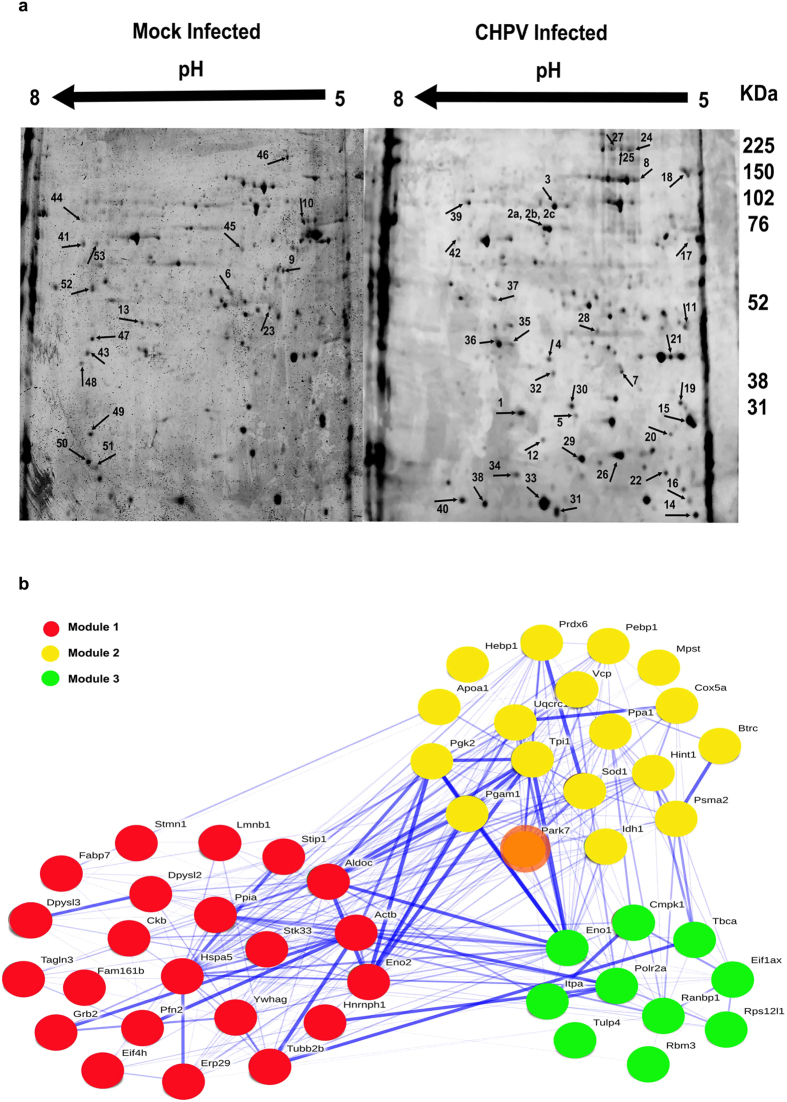
(**a**) Protein was isolated from CHPV infected and mock infected brain samples and subjected to 2-DE. 53 differentially expressed spots were identified and analyzed by MALDI for identification of the proteins. The 2-DE blot images in this figure depict the 53 spots identified from both CHPV and mock infected brain protein samples. (**b**) Modular network formed using 50 proteins identified from MALDI analysis. A PPI network was developed using STRING 10.0 database. The interactions were analyzed using VisualConnectome toolbox in MATLAB that yielded 3 modules with a significant modularity score of 0.17 (against random network modularity score 0.09). The nodes in the figure represent the individual proteins while the edges represent the interactions between the nodes (proteins) based on confidence score. The edges were coloured based on the confidence score (with deep blue correlating to high confidence score and lighter shades of blue to lower confidence scores). Based on the degree centrality parameter DJ-1 node was determined to be the most interactive protein of the interactome model denoted by orange colour.

500 μg of protein from whole brain was isolated from CHPV infected mouse. Along with its mock-infected counterpart the protein sample obtained from CHPV infected mouse brain was subjected to 2-Dimensional Electrophoresis (2-DE) (pH range = 5.0–8.0). 53 differentially expressing protein spots were identified and excised from the gel with the help of PD Quest 2 Dimensional (2D) Analysis software and analyzed with Matrix Assisted Laser Desorption/Ionization (MALDI) (ratio CHPV/mock ≥ |1.5|, using paired Student t-test analysis p < 0.05, spots shown in Fig. 1a). Out of the 53 identified spots 50 proteins were identified through MALDI analysis (3 proteins accession numbers: Q01853, Q9CZ13, P05063 were repetitions) (Tables S.1 and S.2). 17 proteins were identified to have been down-regulated while the rest out of 50 proteins were up-regulated as depicted in Table. S.2.

An interactome model was developed out of the 50 identified proteins with the help of Search Tool for the Retrieval of Interacting Genes/Proteins (STRING) 10.0 online database (http://string-db.org/). Proteins were classified in their respective modules or compartments and arranged in a descending order of “degree centrality” score (Table 1). Modularity is a graph theoretic parameter that defines to what extent a network can be subdivided into subnetworks or modules based on the connectivity of the nodes pertaining to a particular network. The modularity score that ranges between [−0.5, 1] denotes that a network is said to be modular as its corresponding modularity score approaches 1. Another important property of graph theory that we utilized in our network analysis is that of “degree centrality”. “Degree centrality” defines the connectivity of each node in a particular network. A node that is having the highest interaction within a network possess the highest degree centrality score. Modularity score of the interactome was 0.17. 3 modules were identified out of the analysis with Module 1 being the largest consisting of 22 proteins while Modules 2 and 3 consisted of 18 and 10 members, respectively (Fig. 1b & Table 1). Node number 1 (DJ-1) that belonged to the Module 2 was having the highest “degree centrality” score of 1.987 in the interactome (Table 1). This implied that DJ-1 interacted with most of the other proteins in the interactome and holds a strategically important position in the network. Hence DJ-1 was selected for further analysis to determine its role in CHPV life cycle through molecular validation studies^23^,^24^,^25^.

### Scrutinizing the role of DJ-1in CHPV infection in neurons

**Figure 2:**
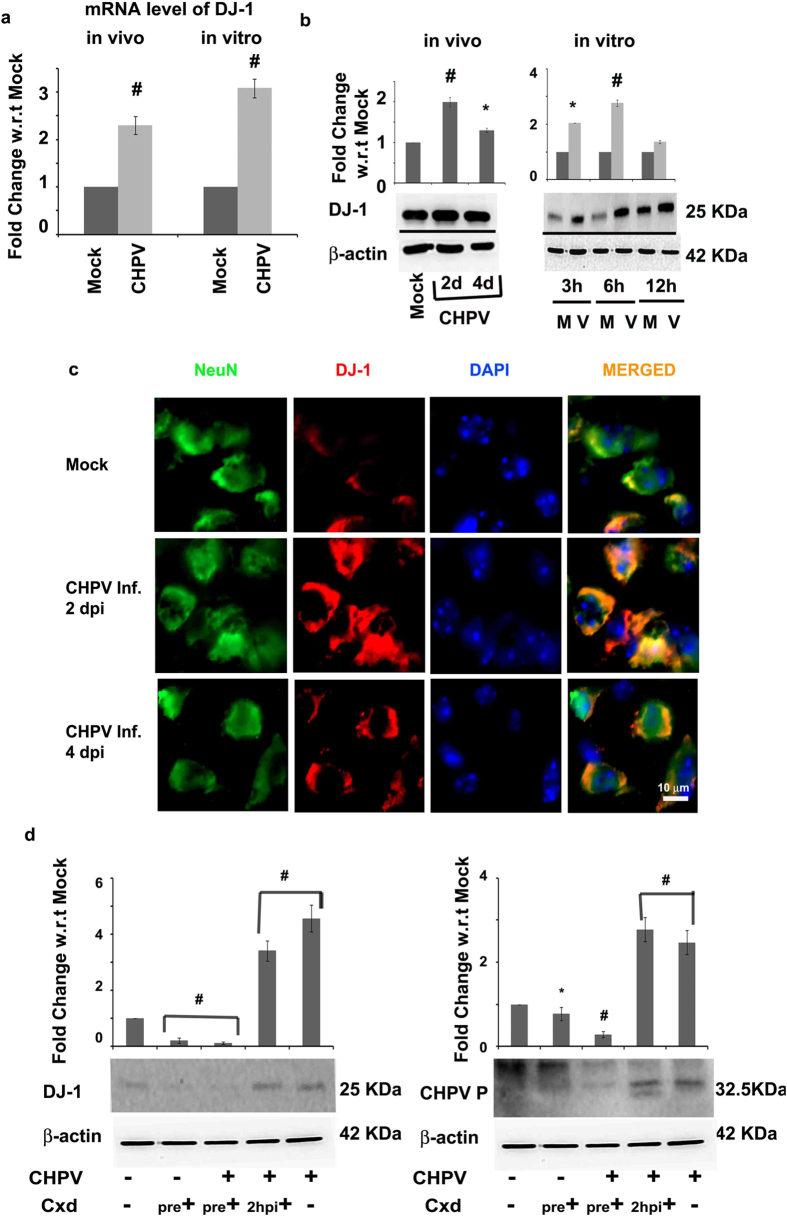
CHPV infection stimulates DJ-1 expression. Representative graph demonstrates the mRNA expression of DJ-1 in mouse brain and Neuro2A cell line. (**a**) Immunoblot images show the expression levels of DJ-1 in both mouse brain and Neuro2A samples observed in a time-dependent analysis. (**b**) In both the cases β-actin was used as a loading control. Co-expression of NeuN (neuronal marker) and DJ-1 was analyzed using immunohistochemistry from mouse brain sections obtained from time-dependent study of CHPV infection in mouse. Representative panel images demonstrate the results of immunohistochemistry analysis. (**c**) Scale = 10 μm. Cxd (100 μg/ml) can effectively shutdown the translational machinery of eukaryotic cells. Expression levels of DJ-1 and CHPV P protein was determined using immunoblot analysis at various time-dependent treatment conditions using proper experimental controls. (**d**) β-actin was used as a loading control. *represents p < 0.05 and ^#^represents p < 0.01. (n = 3).

Significant up-regulation was observed in mRNA levels of DJ-1 in CHPV 4 days post infection (dpi) mouse brain samples (fully symptomatic) and CHPV 12 hours post infection (hpi) neuronal cell line (p < 0.01) (Fig. 2a). A time-based experiment was conducted to monitor the expression of DJ-1 in the course of CHPV infection in both *in vivo* and *in vitro* models of infection through immunoblot analysis. DJ-1 showed an increase in expression in the initial phase of infection in both the cases (p < 0.01) (Fig. 2b). DJ-1 expression was also observed to increase in the mock infected samples of 12 hpi neuronal cell line and densitometric analysis showed a comparable fold change to the CHPV infected samples. This may be due to prolonged exposure to serum free media resulting into oxidative stress in mock samples. However this result does not affect our hypothesis that DJ-1 expression is an early response to the viral infection^26^. Immunohistochemistry analysis concomitantly showed enhanced co-localization of DJ-1 expression in neurons in 2 dpi CHPV infected brain sections in comparison to 4 dpi samples (Fig. 2c).

Cycloheximide (Cxd) has been known to shut down eukaryotic translation^27^. We utilized this chemical to validate our hypothesis that CHPV infection induces DJ-1 expression as an early response. Pre-treating Neuro2A cells with Cxd blocked the viral replication (CHPV P protein) within the cell and DJ-1 expression (p < 0.01) (Fig. 2d and S1). However Cxd treatment 2 hpi of CHPV infection did not have any effect in viral replication or DJ-1 expression.

### Oxidative stress induces DJ-1 expression post CHPV infection

**Figure 3:**
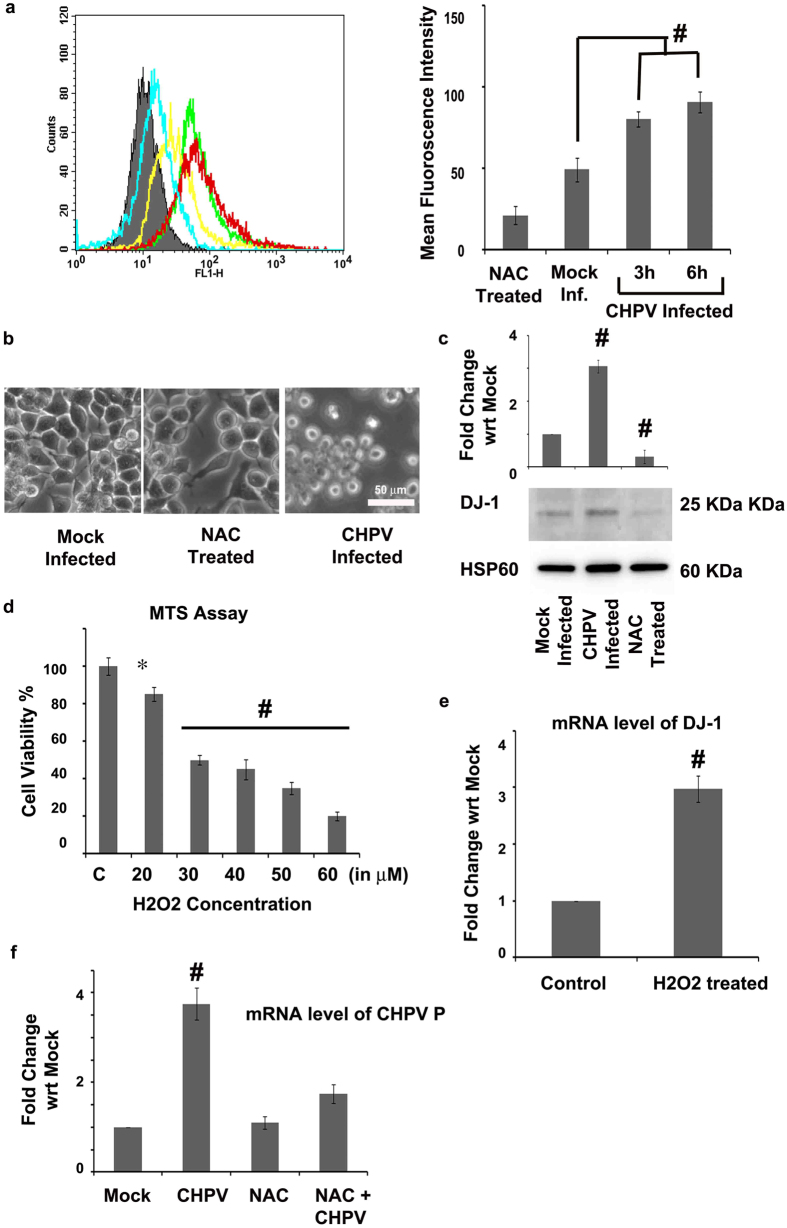
ROS activity triggers DJ-1 expression. DCFDA staining of Neuro2A cells in a time-dependent study of CHPV infection was performed by flow cytometry analysis and the results were quantified as represented by histogram plot and graph. (**a**) The analysis did not involve any gating with a total 10000 event count for each sample. NAC was used as a negative control in the experiment as it is known for its anti-oxidant property. Phase contrast microscopic images compare cell morphology of Neuro2A cells pre-treated with NAC (5 μM) 2 h before CHPV infection against mock infected and CHPV infected cells. (**b**) Scale = 50 μm. Immunoblot image shows the expression levels of DJ-1 in CHPV infected Neuro2A mitochondria 6 hpi using NAC as a negative control in the experiment. (**c**) HSP60 was used as loading control. Working concentration of H_2_O_2_ was determined using MTS assay that reported 50% viability of cells at 30 μM. (**d**) 30 μM of H_2_O_2_ was used to treat Neuro2A cells to monitor the mRNA expression level of DJ-1 (**e**). Representative graph shows mRNA level expression of CHPV P protein in NAC treated Neuro2A cells (**f**). *represents p < 0.05 and ^#^represents p < 0.01. MFI = Mean Fluoroscence Intensity, (n = 3).

2′,7′–dichlorofluorescin diacetate (DCFDA) staining was performed to measure the reactive oxygen species (ROS) level through the course of CHPV infection in Neuro2A cells (p < 0.01) (Fig. 3a). ROS activity increased after 3 hours of infection that explains the triggering of DJ-1 expression at 6 hpi shown in Fig. 2b. To validate the increase in ROS activity is the triggering factor for neuronal apoptosis, cells were pre-treated with N-acetyl cysteine (NAC) before CHPV infection. As evident from Fig. 3b, cells evaded apoptosis in spite of CHPV infection. Increase in ROS activity is generally connected with oxidative stress and mitochondrial dysfunction. DJ-1 translocation to mitochondria was observed in Fig. 3c while in case of NAC treated samples there was no expression of DJ-1 (p < 0.01). In another control experiment to verify that DJ-1 expression is a response to increase in ROS activity, we treated Neuro2A cells with 30 μM of H_2_O_2_ that is known to induce ROS activity (concentration was determined through MTS (3-(4,5-dimethylthiazol-2-yl)-5-(3-carboxymethoxyphenyl)-2-(4-sulfophenyl)-2H-tetrazolium) Assay shown in Fig. 3d). Significant increase in mRNA levels of DJ-1 was observed in H_2_O_2_ treated cells compared to their corresponding control samples (p < 0.01) (Fig. 3e). These evidences collectively strengthen our hypothesis that DJ-1 over-expression is attributed to rise in ROS activity following CHPV infection. Moreover NAC treatment has been observed to reduce CHPV replication that signifies ROS activity and following DJ-1 expression has important roles to play in the viral life cycle (Fig. 3f).

**Figure 4:**
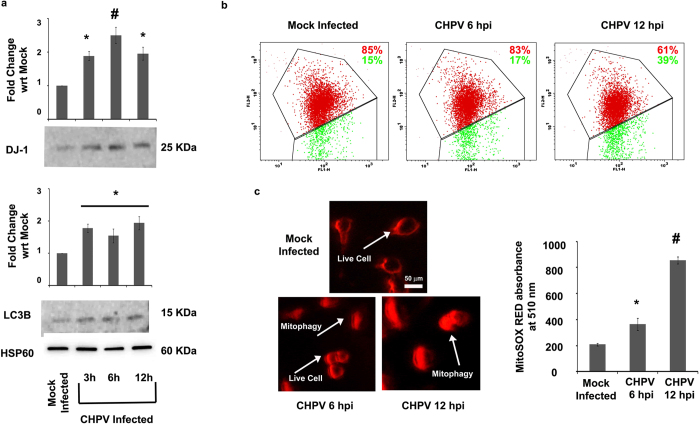
DJ-1 over-expression initiates mitophagy. Immunoblot image shows the expression of DJ-1 and LC3B in mitochondria from a time-dependent study of CHPV infection of Neuro2A. (**a**) HSP60 was used as loading control. JC-1 staining indicates the mitochondrial membrane potential. Representative figures show JC-1 staining with the help of flow cytometric analysis from a time-dependent analysis of CHPV infection of Neuro2A cells. (**b**) The representative plots were a result of gating: Mock Infected = 6075 events, CHPV 6hpi = 5976 events and CHPV 12hpi = 5903 events out of total 10000 events. The percentages mentioned in the panels (percentage of the gated events mentioned previously) quantify the number of cells having majority of mitochondria in polarized state (red) and in depolarized state (green) simply interpreting the oxidative stress condition of the cells. MitoSOX Red staining determines the mitochondrial superoxide production which is a hallmark of mitophagy. The representative image panels show MitoSOX Red staining from a time-dependent analysis of CHPV infection of Neuro2A cells. (**c**) Scale = 50 μm. Corresponding graph indicates the absorbance of MitoSOX Red staining at 510 nm for various time-dependent analysis of CHPV infection of Neuro2A cells. *represents p < 0.05 and ^#^represents p < 0.01. (n = 3).

Oxidative stress has been related to mitochondrial dysfunction in several reports^19^,^28^,^29^,^30^. Cells have an inherent property to remove dysfunctional organelles through a process called autophagy and the term “mitophagy” is coined especially in case of removal of dysfunctional mitochondria through autophagy^31^. In case of CHPV infection of neurons, mitophagy was evidenced with significant over-expression of LC3B (Microtubule-associated protein 1A/1B-light chain 3 conjugated with phosphatidylethanolamine) an autophagic marker (p < 0.05) (Figs 4a and S2). As shown in Figs 4a & S3, DJ-1 over-expression in mitochondria following ROS activity signifies translocation of DJ-1 into mitochondria in response to oxidative stress (p < 0.01). DJ-1 has a regulatory role in maintaining the mitochondrial health. During oxidative stress DJ-1 has been previously reported and evidenced in our experiments, to enter mitochondria to remove dysfunctional mitochondria through mitophagy^19^.

JC-1 (5, 5′, 6, 6′-tetrachloro-1, 1′, 3, 3′-tetraethylbenzimidazol-carbocyanine iodide) is a lipophilic fluorescent cation that distinguishes energized from de-energized mitochondria which infers the health of the cells. JC-1 in monomeric form emits green fluorescence when excited with a light of wavelength ~488nm but in a healthy cell it can get incorporated into the mitochondrial membrane forming J-aggregates due to the physiological membrane potential of mitochondria and its fluorescence shift from green to orange-red. Due to oxidative stress the mitochondrial potential reduces and the J-aggregates disassociate to release JC-1 in monomeric forms into the cytoplasm shifting fluorescence from orange-red to green again^32^. We utilized this molecule to determine the mitochondrial potential through the course of CHPV infection in neurons. The panel images show the maintenance of the potential at 6 hpi indicating that 83% of the total gated cell population is emitting red fluorescence. This is attributed to the regulatory role played by DJ-1 in spite of high ROS generation (Fig. 4b). But, at 12 hpi the mitochondrial potential declines and the healthy cell population declines further to 61% as supported by the expression of LC3B, marking the onset of mitophagy.

The phenomenon of mitophagy following CHPV infection has been further analyzed with the help of a novel fluoroprobe MitoSOX Red. MitoSOX Red is a live-cell permeant probe that selectively targets mitochondria and exhibits bright red fluorescence when oxidized by increasing superoxides which is an indicator of mitochondria undergoing mitophagy^33^. MitoSOX fluorescence increased with the progression of CHPV infection indicating the increase in the number of mitochondria undergoing mitophagy following infection (p < 0.05, p < 0.01) (Fig. 4c). The mock infected cells in the image panel clearly shows no fluorescence inside the cells while the fluorescence went on increasing as the cells infected with CHPV accumulated more number of dysfunctional mitochondria.

### DJ-1 facilitates survival of CHPV in neurons

**Figure 5:**
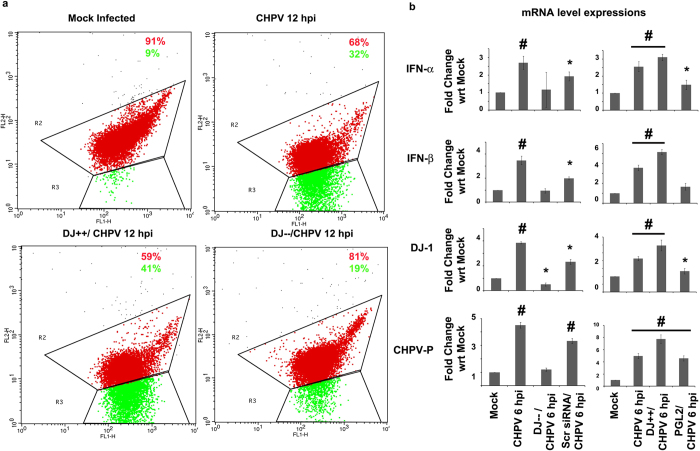
Role of DJ-1 expression in CHPV infected cells. Oxidative stress condition of DJ-1 over-expression and knockdown cells post CHPV 12 hpi were analyzed by JC-1 staining with the help of flow cytometric analysis compared to wild type cells that is illustrated in the representative figures. (**a**) The representative plots were a result of gating: Mock Infected = 8065 events, CHPV 12hpi = 7853 events, DJ++/CHPV 12hpi = 7513 and DJ−−/CHPV 12hpi = 8200 events out of total 10000 events. The percentages mentioned in the panels (percentage of the gated events mentioned previously) quantify the number of cells having majority of mitochondria in polarized state (red) and in depolarized state (green). Representative graphs show mRNA expression levels of IFN- α & β, DJ-1 and CHPV P protein in case of DJ-1 knockdown and over-expression in Neuro2A cells compared to their mock treated samples. (**b**) *represents p < 0.05 and ^#^represents p < 0.01. (n = 3).

We analyzed the mitochondrial health with the help of JC-1 staining in various sample groups: mock infected, CHPV 12 hpi, CHPV infection in DJ-1 over-expressing cells and CHPV infection in DJ-1 knockdown cells. Figure 5a clearly indicates more number of cells are undergoing mitochondrial depolarization in DJ-1 over-expressing cells infected by CHPV compared to other groups, while in absence of DJ-1, cells maintained the population of healthy mitochondria albeit infected by CHPV. Further modulation in DJ-1 expression affected CHPV replication and interferon response (e.g. IFN α and β) in CHPV infected cells as evident from Fig. 5b. It was observed that DJ-1 over-expression supported the CHPV replication indicated by increased mRNA expression level of CHPV P (phosphoprotein) protein in comparison to DJ-1 knockdown cells (p < 0.01). The α/β interferon (IFN) system, consisting of IFN-β and the IFN-α family, represents a crucial defence element of higher organisms that activate both innate and adaptive immunity in response to viral infection^34^. mRNA level expression of both the IFNs were observed to increase significantly in the early phase of infection i.e. 6 hpi in DJ-1 over-expressing cells infected with CHPV in comparison to DJ-1 knockdown cells (p < 0.01).

**Figure 6:**
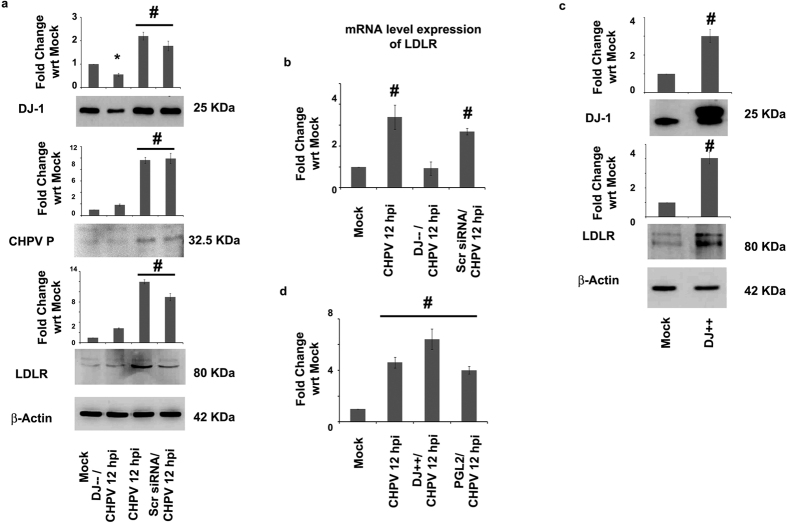
DJ-1 modulates the expression of CHPV P protein and LDLR. Immunoblot image exhibits the expression levels of DJ-1, CHPV P protein and LDLR in case of DJ-1 knockdown Neuro2A cells CHPV 12 hpi along with proper experimental controls. (**a**) β-actin was used as a loading control. mRNA level expression of LDLR showed significant decrease in DJ-1 knockdown cells. (**b**) Immunoblot image shows the expression levels of DJ-1 and LDLR in case of DJ-1 over-expression in Neuro2A cells. (**c**) β-actin was used as a loading control. Supporting the immunoblot image result LDLR mRNA expression was observed to increase significantly in DJ-1 over-expressed cells post CHPV infection (**d**). *represents p < 0.05 and ^#^represents p < 0.01. (n = 3).

Previously we have reported that CHPV perturbs the cholesterol homeostasis in brain by manipulating neurons to import more cholesterol from astrocytes in order to expedite the process of viral assembly^22^. LDLRs mediate this entry of cholesterol within neurons. In the present study we observed a significant decline in the expression levels of both CHPV P protein (p < 0.01) (Fig. 6a) and LDLR (p < 0.01) (Fig. 6ab) in DJ-1 knockdown samples following CHPV infection. On the other hand when DJ-1 was over-expressed in Neuro2A cells, an increase in LDLR was observed (p < 0.01) (Fig. 6c). The over-expression of LDLR was further validated when CHPV was infected in DJ-1 over-expressing cells (p < 0.01) (Fig. 6d). Referring to the previous results we can infer that CHPV infection, ROS generation, mitophagy and DJ-1 expression are not isolated events. Moreover DJ-1 through our experiments and observations may be implicated in playing vital role in CHPV life cycle in neurons.

### DJ-1 modulates the LDLR expression in JEV infection

**Figure 7:**
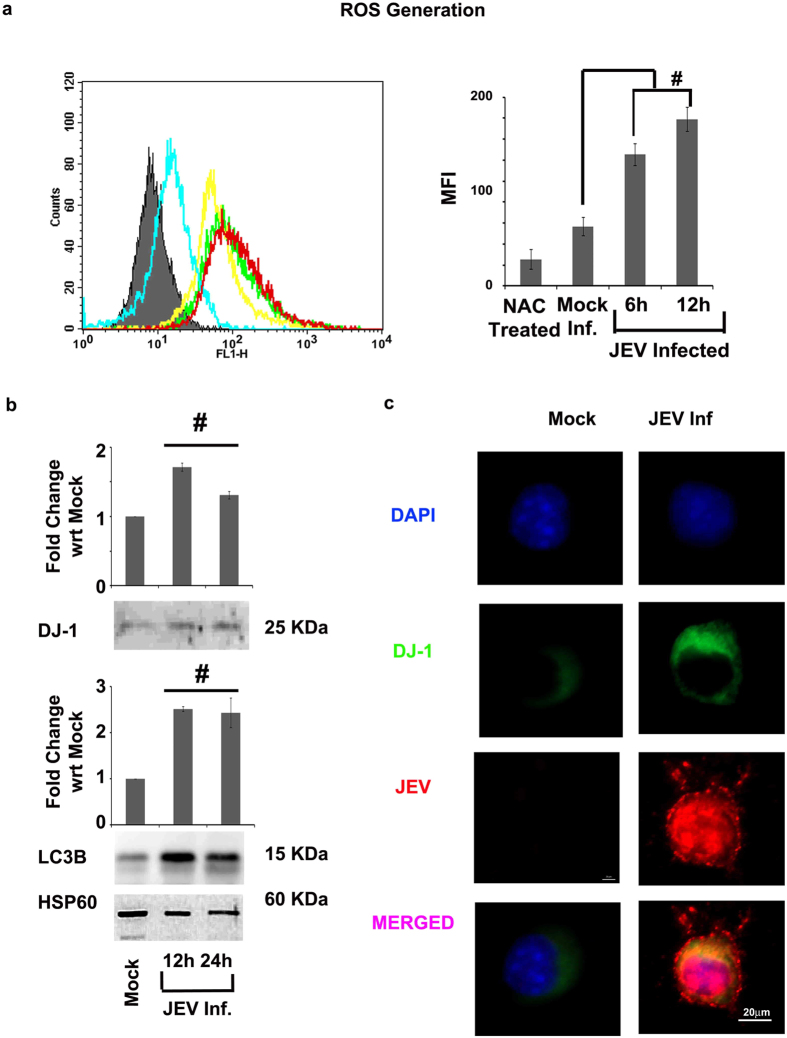
Validation of the proposed hypothesis in JEV infection. DCFDA staining of Neuro2A cells in a time-dependent study of JEV infection was performed by flow cytometry analysis and the results were quantified as represented by histogram plot and graph. (**a**) The analysis did not involve any gating with a total 10000 event count for each sample. N-acetylcysteine (NAC) was used as a negative control in the experiment as it is known for its anti-oxidant property. Immunoblot images in (**b**) exhibits the expression levels of DJ-1 and LC3B in mitochondria from a time-dependent study of JEV infection. HSP60 were used as loading control for the immunoblot analyses. Co-expression of JEV Nakayama (JEV virus marker) and DJ-1 was analyzed using immunocytochemistry from JEV 24 hpi infected Neuro2A cells. Representative panel images demonstrate the results of immunocytochemistry analysis. (**c**) Scale = 10 μm, ^#^represent p < 0.01. MFI = Mean Fluoroscent Intensity. (n = 3).

**Figure 8:**
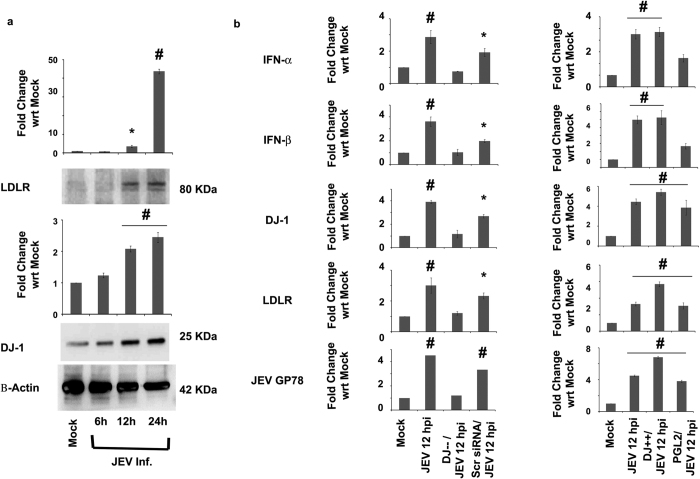
Role of DJ-1 in JEV infected cells. Expression levels of DJ-1 and LDLR are represented in the immunoblot image in a time-dependent study of JEV infection of Neuro2A cells. (**a**) β-actin was used as a loading control. Representative graphs show mRNA expression levels of IFN- α & β, DJ-1, LDLR and JEV GP-78 protein in case of DJ-1 knockdown and over-expression in Neuro2A cells compared to their mock treated samples. (**b**) *represents p < 0.05 and ^#^represents p < 0.01. (n = 3).

The ROS activity showed significant rise in levels in 6 and 12 hpi from culture media of Neuro2A cells infected with JEV (p < 0.01) (Fig. 7a). In response to increase in ROS activity we monitored the mitochondrial DJ-1 and LC3B expression levels by immunoblot analysis that indicated rise in the early phase of infection of JEV (p < 0.01) (Fig. 7b). Similarly as in case of CHPV, DJ-1 showed an enhanced level of expression in the early phase of JEV infection in cytoplasm analyzed through immunocytochemistry (Fig. 7c) and immunoblot (p < 0.01) (Fig. 8a). Immunocytochemistry analysis marks the co-localization of DJ-1 expressing neurons with JEV marker signifying that DJ-1 expression takes place in response to JEV infection. Hence we can infer that both these neurotropic viruses CHPV and JEV induce the increase in ROS activity upon infecting neurons and influences the translocation of DJ-1 from cytoplasm to mitochondria to bring about mitophagy.

LDLR expression levels were observed to increase significantly in a time-dependent infection study for JEV analyzed through immunoblot analysis (p < 0.01) (Fig. 8a,b). As in case of CHPV, DJ-1 was also found to facilitate the replication of JEV as significant increase in the mRNA levels of JEV GP78 was recorded in DJ-1 over-expressing cells compared to RNA extracted from DJ-1 knockdown cells (p < 0.01) (Fig. 8b). Similarly mRNA expression levels of IFN α and β were also observed to increase at an early stage of infection in JEV infected DJ-1 over-expressing cells compared to JEV infected DJ-1 knockdown cells (p < 0.01) (Fig. 8b).

## Discussion

CHPV (negative single stranded RNA virus) and JEV infection (positive single stranded RNA virus) induce neurodegeneration leading to mortality. Our group has established infection models for both these viruses. In case of CHPV infection, 10 day post-natal BALB/c mice developed encephalitis symptoms within 72–96 hpi on inoculation through the intra-peritoneal (i.p.) route and hence succumbed to infection^35^. On contrary, the infection model for JEV was established in adult BALB/c mice (4–6 weeks) that developed encephalitis symptoms 5 dpi inoculating through the same route^36^. Both these viruses are arboviruses and are neurotropic, while JEV belongs to the family *Flaviviridae*, CHPV belongs to the family *Rhabdoviridae*. Albeit the viruses differ in their genomic structures, there are reports that postulate RNA viruses utilize “common” host metabolic pathways for their propagation inside their hosts^37^,^38^.

In our present study we identified DJ-1 to be one of the key players in CHPV pathogenesis through our bioinformatics-based analysis and later on through molecular validation studies. DJ-1 that is implicated to have a neuroprotective role in case of sporadic cases of Parkinson’s disease and other neurodegenerative disease got up-regulated in the viral infected neurons case of CHPV infection. In the initial phase of infection, the neuroprotective role of DJ-1 was observed where it initiated the process of mitophagy in response to increase in ROS activity (Figs 3, 4 and 5). But prolonged ROS activity induced DJ-1 to activate the process of LDLR transcription (Fig. 6). Thus, ROS and DJ-1 together mediate the initiation of infection cascade. LDLR over-expression in neurons infected by viruses stimulated the import of cholesterol from astrocytes as reported in one of our previous publication facilitating the viral assembly and budding in neurons^22^. JEV which is also an enveloped virus was hypothesized to use similar host machinery for deriving its envelope from the host. As per the results obtained from Figs 7 and 8, we proved our hypothesis that DJ-1 also plays an important role in JEV pathogenesis within neurons. Hence DJ-1 may be proposed as an effective common therapeutic target against both CHPV and JEV infection.

As per our findings, cholesterol forms an important component in CHPV life cycle since it helps to form the viral envelope in the assembling and budding phase. Thus in order to search for the host factor/s that influences the perturbation in cholesterol homeostasis and hence neuronal death, whole brain proteome analysis was performed for CHPV infected sample. Proteomic analysis generally provides a huge dataset that becomes difficult to analyze. Modern approaches of systems biology with the incorporation of mathematical modelling have been useful in analyzing such “big data”^39^. Through our proteomic analysis we identified 50 proteins which got differentially expressed in case of CHPV infection (Fig. 1). Subsequently, we utilized the measures of graph theory to analyze and quantitate the role of specific protein modules in the interactome formed by 50 identified proteins (nodes).

Degree centrality is a measure in network analysis that defines the connectivity and the importance each node is having in a particular network. Centrality measure has been previously utilized in prioritization of proteins (i.e. ranking the nodes/proteins in a network depending on their strategic importance within that network) in case of complex PPI networks^40^. Conventionally centrality has been classified into: (1) degree centrality, (2) closeness, (3) betweenness and (4) eigen centrality. We have previously established that degree centrality is an effective measure in protein prioritization and has been observed to be consistent over larger networks^17^. Hence the “degree centrality” parameter was chosen to quantitate the contribution of a particular node within a network. Table 1 showed that DJ-1 was having the highest degree centrality score of 1.987 in the interactome. We explored the potential role of DJ-1 in the context of CHPV infection in neurons since CHPV selectively replicates in neurons of Central Nervous System (CNS)^22^.

DJ-1 has been implicated in several neurodegenerative diseases especially in the sporadic cases of onset of Parkinson’s disease^41^. Enhancement of DJ-1 in cells in case of neurodegenerative diseases have been reported to be in response to an increase in oxidative stress^28^. Complying with the previous reports oxidative stress was evidenced in both CHPV and JEV infection by increase in ROS activity. Treatment with NAC reversed the effect of ROS activity evading neuronal apoptosis and DJ-1 over-expression (Fig. 3). Again treatment with Cxd (that blocks the translational machinery of the cells) clearly showed that CHPV replication is correlated with expression of DJ-1 (Fig. 2). Hence the results comprehensively prove that the process of viral replication stimulates the ROS production and up-regulation of DJ-1.

Several reports have been published over the years that associate the process of viral replication and ROS production in cells but the exact mechanism is still elusive^42^. RNA viruses on entering the cell try to hack the host metabolic machinery and in the process interfere with the cell’s ROS system. Mitochondrial DNA (mtDNA) encodes for 13 polypeptides, 2 ribosomal RNA (rRNA) and 22 transfer RNA (tRNA)^43^. Enhanced ROS production directly targets the mtDNA and affects the production of all these byproducts that contribute to the proper functioning of the electron transport chain leading to mitochondrial dysfunction. While other theories state that manipulation of protein folding and assembly machinery of Endoplasmic Reticulum (ER) lead to ER stress perturbing the calcium homeostasis. Calcium ion (Ca^2+^) under normal physiological condition activates the mitochondrial metabolism but in altered condition switch from a physiological beneficial process to a cell death signal^44^. Hence mitochondrial dysfunction or a newly coined term “mitophagy” is quite pronounced in case of uncontrolled ROS production.

Mitophagy was evidenced in case of CHPV infection in Fig. 4 that showed over-expression of autophagy marker LC3B (whose role was also monitored in case of JEV infection), decrease in mitochondrial potential and MitoSOX Red fluorescence at 12 hpi. DJ-1 has been previously reported to associate with Parkin and Pink-1 for regulating mitochondrial function and mitophagy in response to oxidative stress^18^. Our present result shows that DJ-1 up-regulates at 6 hpi and induces mitophagy at an early phase of infection (JC-1 staining Figs 3, 4, 5). Hence the prominent role played by DJ-1 in CHPV and JEV infection proved through molecular studies validates the selection of DJ-1 through graph theoretic analysis.

Ablation of DJ-1 enhances the ROS activity and induces a faster rate of mitophagy^45^. Contrary to the reports^46^,^47^, knockdown of DJ-1 in case of CHPV infection alleviated neuronal health and decreased the number of dysfunctional mitochondria while DJ-1 over-expression stimulated mitophagy (Fig. 5a). Surprisingly the replication of both CHPV and JEV was found to be affected by DJ-1 knockdown and potentiated by DJ-1 over-expression (Figs 5b and 8b). In addition to this, interferon response to the viral infection was observed to get modulated by DJ-1, in case of both these viruses (Figs 5b and 8b). Moreover as discussed earlier, Cxd pre-treatment of cells, blocked CHPV P protein expression along with DJ-1. Hence it may be hypothesized that DJ-1 must be playing a vital role in both CHPV and JEV life cycle that was facilitating the survival of these neurotropic viruses within neurons.

Following our previous finding that LDLR facilitated the import of cholesterol into neurons from astrocytes which helps in the propagation of CHPV, LDLR expression got affected by DJ-1 expression (Fig. 6). While, over-expression of DJ-1 following CHPV and JEV infection enhanced LDLR expression, knockdown of DJ-1 diminished the same (Figs 6 and 8). Hence through our observations in both the viruses it may be hypothesized that DJ-1 expression modulates LDLR expression that further affects the propagation of the neurotropic viruses. The mechanism of DJ-1 modulation of LDLR has been further discussed in the Supplementary Information.

**Figure 9:**
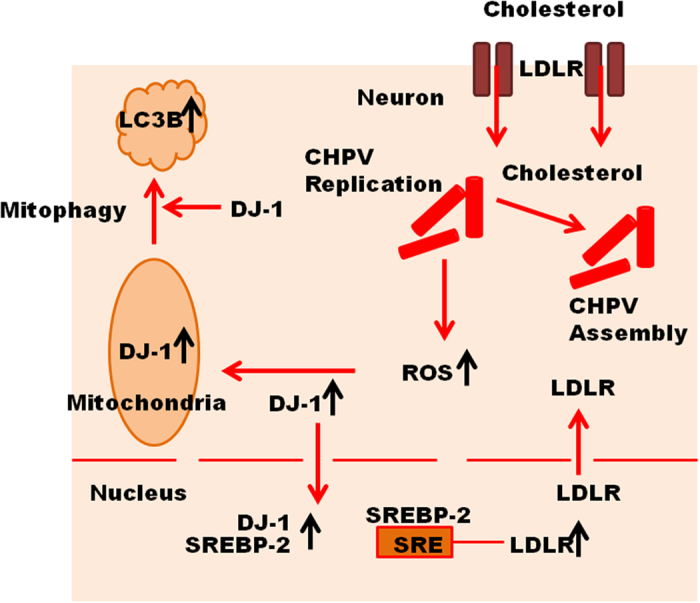
Schematic diagram representing the possible role of DJ-1 in CHPV infection. CHPV replication stimulates the release of ROS in neurons. Increase of ROS in cytoplasm induces the over-expression of DJ-1 that translocates in mitochondria to induce mitophagy signified by the expression of LC3B. DJ-1 over-expression in response to ROS activity influences migration of both DJ-1 and SREBP-2 into nucleus. DJ-1 co-activates SREBP-2 to bind to the SRE binding site of LDLR promoter and regulate the expression of the later. LDLR over-expression favours import of cholesterol into neurons facilitating CHPV assembly.

In summary, DJ-1 plays a critical role in pathogenesis of both CHPV and JEV infection in neurons. Viral replication in neurons triggers ROS activity in response to which DJ-1 over-expresses in the initial phase of infection to prevent inflammation. Thus, DJ-1 has a neuroprotective role attributed to its anti-oxidant property. In the initial phase of infection, DJ-1 induces mitophagy in response to viral infection. Prolonged infection causes uncontrolled release of ROS in response to which DJ-1 activity also increases. DJ-1 triggers the transcription of LDLR. As reported previously, enhanced expression of LDLR increases the import of excess cholesterol in neurons from astrocytes that is utilized by CHPV for its envelope formation. The entire mechanism has been described in a schematic representation in Fig. 9. Another aspect that comes up from our research is that both JEV and CHPV adapt a similar mechanistic approach in neuronal infection. It would be of interest to explore whether other neurotropic RNA viruses also adapt a similar approach.

## Materials and Methods

### Ethics Statement

All animal experiments were approved by the Institutional Animal and Ethics Committee (IAEC) of the National Brain Research Centre (approval no. NBRC/IAEC/2012/70). The animals were handled in strict accordance with good animal practice as defined by the Committee for the Purpose of Control and Supervision of Experiments on Animals (CPCSEA), Ministry of Environment and Forestry, Government of India.

### Virus and cells

CHPV (strain no. 1653514 isolated from human patient in Nagpur, 2003) was propagated in Vero cell line^22^,^48^. The titer of the virus was found to be 3 × 10^5^ plaque forming units (pfu)/ml. Neuro2A and Vero cells (Vero cells were a kind gift from Prof. Debiprasad Sarkar, Dept of Biochemistry, University of Delhi South Campus, India) were grown at 37 °C in Dulbecco’s Modified Eagle Medium (DMEM) supplemented with 3.5% sodium bicarbonate, 10% fetal bovine serum (FBS) and penicillin/streptomycin (Sigma Aldrich, St. Louis, MO, USA).

GP78 strain of JEV was propagated in suckling BALB/c mice and their brains were harvested when pathological symptoms were observed. Virus titrations were conducted and quantified as described earlier^49^.

### Animal treatment

BALB/c mouse pups of 10 post natal days were used for the experiments and always kept with mother for milk feeding. The animals were divided into two groups- mock and CHPV-infected (irrespective of sex since CHPV infects without any sexual bias). CHPV group was injected with 50 μl of virus (approximately 1.5 × 10^4^ pfu) while the mock infected animals were injected with a similar volume of Phosphate buffer solution (PBS) through i.p. route. CHPV-infected animals succumbed by 76−92 hpi. Animals of mock infected group were also sacrificed at the same time. Brains were excised after repeated transcardial perfusion with ice-cold 1X PBS followed by tissue fixation using 10% paraformaldehyde (PFA) (Sigma Aldrich, St. Louis, MO, USA).

### Infection and treatment of Neuro2A cells

Neuro2A cells were cultured in serum containing medium till 70 to 80% confluence, followed by differentiation in serum-free medium. Cells were then either mock infected (PBS of equal volume as that of virus) or infected with CHPV at a multiplicity of infection (MOI) of 0.1 and kept in incubation for 2 hours. Post-infection, cells were washed thrice with sterile 1× PBS to remove non-internalized virus and were incubated for different time periods in serum-free medium. JEV infection was also applied in a similar manner with a MOI of 5.

#### N-acetylcysteine (NAC) Treatment

5 μM of NAC (Sigma Aldrich, St. Louis, MO, USA) was administered in the culture plates of Neuro2A, 2 hours before infecting with virus.

#### Cycloheximide (Cxd) Treatment

100 μg/ml of Cxd (Sigma Aldrich, St. Louis, MO, USA) was administered both prior and after two hours of CHPV infection to Neuro2A separately to observe the effect.

#### H_2_O_2_ Treatment

30 μM of H_2_O_2_ (Sigma Aldrich, St. Louis, MO, USA) (determined through MTS assay as described previously^35^) was administered to Neuro2A to induce intracellular ROS generation.

#### Transfection of Neuro2A

10 μM of DJ-1 esiRNA (Endoribonuclease-prepared siRNA) (Sigma Aldrich, St. Louis, MO, USA) or 1 μg of pRK5-mouse-DJ1-HA (was a gift from Dr. Mark Cookson, National Institute on Aging, National Institutes of Health, Bethesda)^50^ plasmid was used for transfection using Lipofectamine (Invitrogen, Carlsbad, CA, USA) according to the manufacturer’s protocol. Briefly, Neuro2A cells were seeded and maintained in sets of three at 37 °C and 5% CO_2_ and when the cells were 70% to 80% confluent, they were transfected in Opti-MEM (Invitrogen, Carlsbad, CA, USA) for 6 hours after which fresh 5% DMEM was added to the cells for 24 hours. While DJ-1 esiRNA was used to knockdown the expression of DJ-1, pRK5-mouse-DJ1-HA was used to over-express DJ-1 in Neuro2A cells.

### Plaque Assay for determining pfu/ml of CHPV and JEV

Briefly, Vero cells were seeded in six-well plates to form confluent monolayer. Cell monolayer were inoculated with 10-fold serial dilutions of supernatant samples made in Minimal Essential Medium (MEM) containing 1% FBS and incubated for 1 h at 37 °C with occasional shaking. The inoculum was removed by aspiration and the monolayers were overlaid with MEM containing 4% FBS, 1% low-melting-point agarose and a cocktail of antibiotic–antimycotic solution (Gibco, Invitrogen Corporation, Grassland, NY, USA) containing penicillin, streptomycin, and amphotericin B (Sigma Aldrich, St. Louis, MO, USA). Plates were incubated at 37 °C for 18−24 h until plaques became visible. To allow counting of the plaques, the cell monolayer was stained with crystal violet after fixing the cells with 10% PFA.

### Protein extraction from brain tissue and neuroblastoma cell line

The brain tissues were thawed on ice and homogenized using Ultra-turrax T8 homogenizer (IKA-Werke GmbH & Co. KG, Germany) in solubilization buffer containing 8M urea, 2% (w/v) CHAPS (3-[(3-cholamidopropyl)dimethylammonio]-1-propanesulfonate), 0.2% sodium orthovanadate and 1 × concentration of protease inhibitor cocktail (Sigma Aldrich, St. Louis, MO, USA). The supernatant was collected by centrifugation and subjected to three pulses of sonication on ice followed by centrifugation at 20,000 × g for 30 min at 4 °C for the recovery of total soluble proteins. Protein extraction from Neuro2a cells was carried out in a similar manner. The rest of the extraction procedure was done according to ref. 51.

#### Mitochondrial protein extraction from neuroblastoma cell line

After the respective incubation periods as per the experiments, cells were harvested with the help of 1x PBS. From there on mitochondrial protein extraction was performed as per the manufacturer’s instruction. (Mitochondria Isolation Kit for Cultured Cells, Cat. 89874, ThermoFisher).

### Nuclear protein extraction from neuroblastoma cell line

For nuclear extracts, Neuro2A cells were collected with 1X PBS and harvested by spinning at 2000 rpm for 5 min. The cells were resuspended in 400 μl of cold buffer A (10 mM HEPES pH 7.9, 10 mM KCl, 0.1 mM EDTA, 0.1 mM EGTA, 1 mM DTT and 0.5 mM PMSF) (Sigma Aldrich, St. Louis, MO, USA) and then kept on ice for 15 min. 15 μl of nonionic surfactant, IGEPAL CA 630 (Sigma Aldrich, St. Louis, MO, USA) was then added and vortexed vigorously. The cells were then pelleted for 1 min at 10,000 g and the recovered pellet was re-suspended in 50 μl of ice cold buffer B (20 mM HEPES pH 7.9, 400 mM KCl and 1 mM EDTA) and subjected to gentle shaking for 15 min at 4 °C. The suspended cells in Buffer B were again pelleted at 4 °C and the supernatant having the nuclear protein was collected and estimated by bicinchoninic assay (BCA) method.

### Two dimensional gel electrophoresis (2-DE)

2-DE was performed as described earlier. The protein pellet from the brain tissue (extraction procedure as mentioned above) was re-suspended in sample rehydration buffer (8 M urea, 2% w/v CHAPS, 15 mM DTT and 0.5% v/v IPG buffer pH 3–10). The iso-electric focusing was performed using immobilized pH gradient (IPG) strips (Bio-Rad, CA, USA). IPG strips of 7 cm size with a pH range from 5–8 were used for all the experiments. For the first dimension 500 μg of protein samples in 150 μl of rehydration solution was used to passively rehydrate IPG strips overnight. The proteins were then focused for 10000 VHr at 20 °C under mineral oil on a Protean i12™ IEF Cell (Bio-Rad, CA, USA). After focusing, the strips were incubated for 10 min, in 2 ml of equilibration buffer I (6 M urea, 30% w/v glycerol, 2% w/v SDS and 1% w/v DTT in 50 mM Tris/HCl buffer, pH 8.8) followed by equilibration buffer II (6 M urea, 30% w/v glycerol, 2% w/v SDS and 4% w/v iodoacetamide in 375 mM Tris/HCl buffer, pH 8.8). After the equilibration steps the strips were transferred to 10% SDS-PAGE for the second dimension by the method of Blackshear^52^.

### Protein visualization and image analysis

Protein spots were visualized by staining with Coomassie Brilliant Blue G-250. Gel images were captured by LI-COR odyssey infra red imager (LI-COR Biosciences, NE, USA). Four biological replicates each with two analytical replicate (n = 8) images per dataset (mock infected versus CHPV infected) were used for automatic spot detection using the PD Quest 2D Analysis software (Bio-Rad, CA, USA). Spot intensities were normalized by total valid spot intensities and mean of values from duplicate analytical gels from four biological replicates were subjected to paired *t-*test analysis. Protein spots showing altered expression between mock and CHPV infected groups (|ratio| ≥ 1.5, *p* ≥ 0.05) were marked and excised.

### Generation of peptides and their extraction from gel spots

The gel spots were washed in 50 mM NH_4_HCO_3_/acetonitrile (1:1) solution to remove the coomassie stain. Reduction of the protein in the gel pieces were performed by incubating them in 10 mM DTT (dissolved in 50 mM NH_4_HCO_3_ solution) for 45 min at 56 °C. Subsequent alkylation was done by incubating the gel pieces with 55 mM iodoacetamide (dissolved in 50 mM NH_4_HCO_3_ solution) for 30 minutes in the dark. The gel pieces were then incubated with 50–100 ng Trypsin overnight at 37 °C. The peptides formed after digestion, were then extracted in Acetonitrile solution containing 0.1% Tri-Fluoro Acetic acid.

### Mass spectrometric peptide analysis

MALDI-TOF (MALDI-Time of Flight) was performed using Bruker ultra flex extreme. The instrument parameters were set as follows: detector, reflector mode; accelerating voltage, 25 kV; delay time, 1 μs; laser intensity, 2500. Acquisition was made in the range m/z 700–3500 Da. A total of 500 shots were performed per spectrum, and 20–25 spectra were accumulated per sample to increase the signal to noise ratio. Spectra were acquired in the positive ion mode. A volume of 2 μL of digested sample was mixed with 2 μL of saturated hydroxylcinnamic acid solution and from this mixture 1 μL was deposited onto a stainless steel MALDI sample target and air-dried. Searches were performed against the SWISS PROT protein sequence database allowing for up to 100 ppm error tolerance and up to one missed trypsin cleavage site. The carbamido-methylation of cysteines and methionine oxidation were selected as variable modifications during the search. MS/MS process was done by using LIFT method. The MS & MS/MS spectra were combined & searched using MASCOT against SWISS PROT protein sequence database allowing for up to 0.1 ppm error tolerance.

### Generation of meta-network

The 50 proteins identified through MALDI analysis were queried in the STRING 10.0 querying for 0 interacting partners (since we were looking for developing an interactome out of the 50 identified proteins) as an output from the *Mus musculus* database. The STRING 10.0 software defines significance of the interactions between various queried proteins in terms of confidence score. This confidence score is an empirical score defined by the number of citations and experimental evidence for a particular interaction. The minimum (0.15) confidence score in the database was chosen in our case since we tried to obtain all possible interactions between the 50 nodes queried for building the network. Hence the edges that connect the nodes carry certain weightage score based on the confidence of the interaction. The deep blue shade corresponds to highest confidence and lighter shades of blue for lower confidence scores. The interactome was designed based on active prediction methods as neighbourhood, gene fusion, co-occurence and co-expression.

### Graph theoretic analysis

The adjacency matrix for graph theoretic analysis was created from the interactome as derived from the STRING 10.0 database. Visual Connectome analysis tool box in MATLAB was used to compute the modularity score and the degree centrality of all the nodes^53^.

### Degree Centrality

Degree centrality is the property that defines the connectivity of particular node with other nodes of the same network. This means the higher number of connections of a particular node with other nodes in a network, higher is its degree centrality. The node with the highest degree centrality is the one through which maximum edges pass.

Degree centrality of a vertex *v*, for a given graph G = (V, E) with |V| vertices and |E| edges is defined as *Z*(*v*) = *deg*(*v*).

#### Modularity

Modularity score is used to measure the community structure within a network. The value of modularity ranges between [−0.5, 1) with 0 and negative values meaning a network with randomly assigned edges to positive values indicating highly communal structure.

The detailed algorithm is mentioned in ref. 17.

### Immunoblotting

Protein isolation from both tissue and cells were done as mentioned earlier. Primary antibodies against DJ-1, LC3B (Abcam, MA, USA), SREBP-2 (Santa Cruz, CA, USA), CHPV P^22^ at 1:1000 and LDLR (Abcam, MA, USA) at 1: 500 dilutions, were used for studying the expression levels of respective proteins. The blots were processed for development using chemiluminescence reagent (Millipore, MA, USA). The images were captured and analyzed using Chemigenius Bioimaging System (Syngene, Cambridge, UK). To determine equivalent loading of samples the blots were stripped and reprobed with β-Actin (Sigma Aldrich, USA) (whole cell), HSP60 (Abcam, MA USA) (mitochondria), PCNA (Cell Signalling, MA, USA) (nuclear).

### Immunohistochemistry/cytochemistry

Immunohisto/cytochemistry was performed following previous published protocol^35^. Primary antibodies against neuronal marker NeuN (Chemicon, CA, USA), DJ-1 (Abcam, MA, USA) at 1:500 and JEV Nakayama (Chemicon, CA, USA) at 1:100 dilutions, were used to check the expression levels of the respective proteins. For the Mito-tracker (a kind gift from Dr. Ellora Sen, NBRC) experiment, cells were incubated with 500 nM of Mitotracker probe prepared in prewarmed (37 °C) serum free media and incubated for 45 min at 37 °C. After incubation, staining media was replaced with fresh pre-warm PBS and fixed with PFA. In order to study the expression of DJ-1 and LC3B co-localization within mitochondria, fixed cells were probed with the required antibodies as per established protocol. Post staining sections were mounted with DAPI (Vector Laboratories Inc, CA, USA), observed under a Axio Observer.Z1 Fluorescence microscope (Carl Zeiss, Germany) and images were captured with AxioCam MRm. Image acquisition was done with Zen pro 2011. We used Adobe Photoshop 7.0 for adjusting the contrast and brightness of the images.

### Quantitative PCR

Total cellular RNA from whole brain tissue and Neuro2A was extracted using Tri reagent (Sigma Aldrich, USA). cDNA synthesis was performed using advantage RT-PCR Kit (Clontech, Mountain view, USA) and run on a ABI Prism 7500 sequence detection system (Applied Biosystems, USA). Primers used are enlisted in Table 2. The results were normalized with GAPDH.

### Measurement of reactive oxygen species (ROS)

We examined the effects of CHPV and JEV on Neuro2A cells by determining the levels of ROS. Intracellular ROS generation in mock and virus infected cells was assessed using the cell permeable, non-polar hydrogen peroxide-sensitive dye 5-(and-6)-chlromethyl-2′, 7′-dichlorodihydrofluorescein diacetate (DCFDA) (Sigma Aldrich, MO, USA) and the mean fluorescent intensities (MFIs) were measured on the FL-1 channel on a fluorescence-activated cell sorting (FACS) Calibur flow cytometer (Becton Dickinson, Franklin Lakes, NJ, USA) as described previously^54^.

### Measurement of mitochondrial activity

We examined the effect of CHPV on Neuro2A mitochondria in various phases of infection and in DJ-1 knockdown cells. The mitochondrial activity was measured using JC-1 staining (5,5′,6,6′-tetrachloro-1,1′,3,3′-tetraethylbenzimidazol-carbocyanine iodide) (a kind gift from Prof. S. K. Sharma and Prof. Pankaj Seth, NBRC). After the incubation period was over 2 μM JC-1 was administered in the culture medium and cells were incubated for 15 min at 37 °C, 5% CO_2_. Cells were harvested with phosphate-buffered saline (PBS) and analyzed on a fluorescence-activated cell sorting (FACS) Calibur flow cytometer (Becton Dickinson, Franklin Lakes, NJ, USA) using 488 nm excitation with 530 nm and 585 nm bandpass emission filters. Cells were gated and analyzed using Cell Quest Pro software (Becton Dickinson, Franklin Lakes, NJ, USA). The details of the gatings applied are mentioned in the figure legends of corresponding images.

### Luciferase Assay

Luciferase reporter gene constructs pLDLR-Luc mutSRE was a gift from Axel Nohturfft (Addgene plasmid # 14945)^55^ was used, containing human Sterol Regulatory Element (SRE) binding site promoter region of LDLR (−335 to +3 bp) cloned upstream of the luciferase reporter gene in the pGL2-basic vector. The SRE sequence had point mutation at ATCACCCCAC. Using primers: Forward- GGTGAAGACATTTGAAAAT-CACCCCACTGCAAACTCC, Reverse- GGAGTTTGCAGTGGGGTGATTTTCAAA-TGTCTTCACC and Dpn1 (NEB, UK) mutation was performed to get the wild type sequence ATAACCCCAC (pLDLR-Luc SRE). Since the SRE binding site is conserved through human and mouse we transfected both pLDL-Luc mutSRE and pLDL-Luc SRE into Neuro2A cells separately and infected the cells with CHPV to check the luciferase activity^56^. The luciferase assay was carried out using the luciferase assay kit (Promega, USA) according to the manufacturer’s protocol. The reading was taken using a Sirius single tube luminometer (Berthold Detection Systems GmBH, Germany). The luciferase units were measured as relative luciferase units and these values represented as fold change with respect to pGL2-basic vector (taken as control) reading.

### MitoSOX Red Staining

Mitophagy was determined using fluorescent dye MitoSOX Red Mitochondrial Superoxide Indicator (Life Technologies-Invitrogen). Briefly, cells were seeded in 35 mm culture and infected with CHPV. After the incubation period was over plates were incubated with 2 *μ*M MitoSOX for 20 min. Images of fluorescently labeled cells were captured by inverted fluorescence microscope (Nikon Eclipse Ti-S, Japan), using a Rhodamine filter. Absorbance was measured at absorption/emission maxima: ~510/580 nm in Sirius single tube luminometer (Berthold Detection Systems GmBH, Germany).

### Statistical Analysis

Data were compared between groups using paired t-test using GrapPad Prism 6 software. All data were considered to be statistically significant if p value < 0.05 represented as *and highly significant if p value < 0.01 represented as #.

## Additional Information

**How to cite this article**: Ghosh, S. *et al.* Network analysis reveals common host protein/s modulating pathogenesis of neurotropic viruses. *Sci. Rep.* **6**, 32593; doi: 10.1038/srep32593 (2016).

## Supplementary Material

Supplementary Information

## Figures and Tables

**Table 1:** Protein Distribution & Degree Centrality Score from Modularity Analysis.

Module 1		Module 2		Module 3	
Protein Symbol	Degree Centrality (Z score)	Protein Symbol	Degree Centrality (Z score)	Protein Symbol	Degree Centrality (Z score)
ACTB	1.69249	DJ-1	1.987414	Eif1ax	1.769303
HSPA5	1.69249	SOD1	1.926947	Ranbp1	0.884652
ENO2	1.306135	Ppa1	1.179751	ENO1	0.884652
PPIA	1.085096	TPI1	1.179751	ITPA	0.442326
YWHAG	0.864058	PRDX6	0.674144	Cmpk1	0
ALDOC	0.864058	UQCRC1	0.674144	TBCA	−0.44233
DPYSL3	0.200944	PGAM1	0.674144	Rps12l1	−0.44233
TUBB2B	0.200944	COX5A	0.168536	RBM3	−0.44233
DPYSL2	0.200944	PEBP1	0.168536	Polr2a	−1.32698
VIME	−0.02009	VCP	0.168536	TULP4	−1.32698
ERP29	−0.02009	PGK2	0.168536		
CKB	−0.24113	HINT1	0.168536		
TAGL3	−0.24113	PSMA2	0.168536		
STIP1	−0.46217	IDH1	−0.33707		
LMNB1	−0.68321	APOA1	−1.34829		
FABP7	−0.68321	BTRC	−1.60109		
Pfn2	−0.68321	Mpst	−1.85389		
Eif4h	−0.68321	HEBP1	−1.85389		
HNRH1	−0.90425				
STMN1	−1.12529				
GRB2	−1.12529				
F161B	−1.7884				

**Table 2:** Primer List.

Gene Name	Forward Sequence	Reverse Sequence
DJ-1	TTTATCTGAGTCGCCTATGG	CTCTCTGAGTAGCTGTAGTG
SOD-1	CACTCTAAGAAACATGGTGG	GATCACACGATCTTCAATGG
IFN-α	ATT GGC TAG GCT CTG TGC TTT	AGG GCT CTC CAG ACT TCT GC
IFN-β	TTG CCA TCC AAG AGA TGC TC	TCA GAA ACA CTG TCT GCT GG
CHPV P Protein	CACAGCTTGGAACCTTCTCC	TGACCGGGTTGAGGATTGGC
JEV GP78	TTG ACA ATC ATG GCA AAC G	CCC AAC TTG CGC TGA ATA A
LDLR	CGGCCCTGGCAGTTCTGTGG	CGCGGATCTGATGCGTCGCC
