# Supplementary material for: Network analysis reveals common host protein/s modulating pathogenesis of neurotropic viruses
Source: Sci Rep. 2016 Sep 1;6:32593. doi: 10.1038/srep32593 (PMC5007645; doi:10.1038/srep32593)
Supplement: Supplementary Information [file srep32593-s1.pdf]

Network analysis reveals common host protein/s modulating pathogenesis of neurotropic viruses

Sourish Ghosh[1], *Sriparna Mukherjee*[1], *Nabonita Sengupta*[1], *Arunava Roy*[2^], *Dhritiman Dey*[2], *Surajit Chakraborty*[1], *Dhrubajyoti Chattopadhyay*[2], *Arpan Banerjee*[1], *Anirban Basu*[1¥]

Running Title: *Graph theory and neurotropic viruses*

Keywords: CHPV, JEV, DJ-1, graph theory, proteomics, LDL receptor

Abstract: 197 words, Main Text: 4161 words, Figures: 8, Tables: 4, Graphical Abstract: 1

1. National Brain Research Centre, Manesar, Haryana-122051, India
2. Department of Biochemistry, University of Calcutta, Kolkata- 700019, India
3. ^Present affiliation: H. M. Bligh Cancer Research Laboratories, Department of Microbiology and Immunology, Chicago Medical School, Rosalind Franklin University of Medicine and Science, North Chicago, Illinois, United States of America

¥To whom correspondence should be addressed: Anirban Basu, National Brain Research Centre, Manesar, Haryana-122051, India. Tel.: 91-124-2845225, Email: anirban@nbrc.ac.in

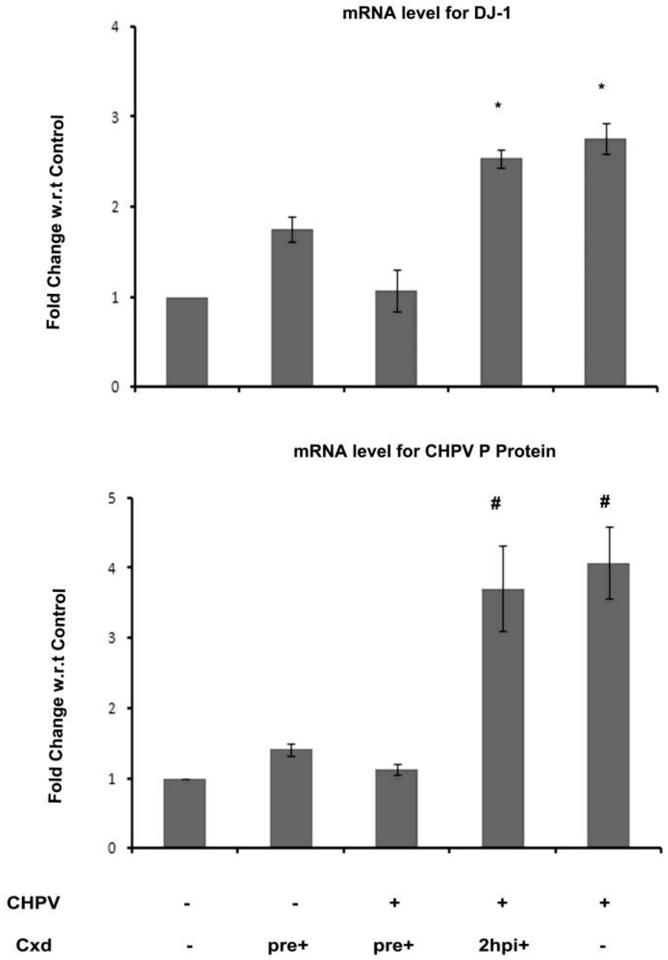

Fig. S1. Cycloheximide (100µg/ml) can effectively shutdown the translational machinery of eukaryotic cells. mRNA expression levels of DJ-1 and CHPV P protein was determined using qPCR analysis at various time-dependent treatment conditions using proper experimental controls (D). * represents $p < 0.05$ and # represents $p < 0.01$. (n=3)

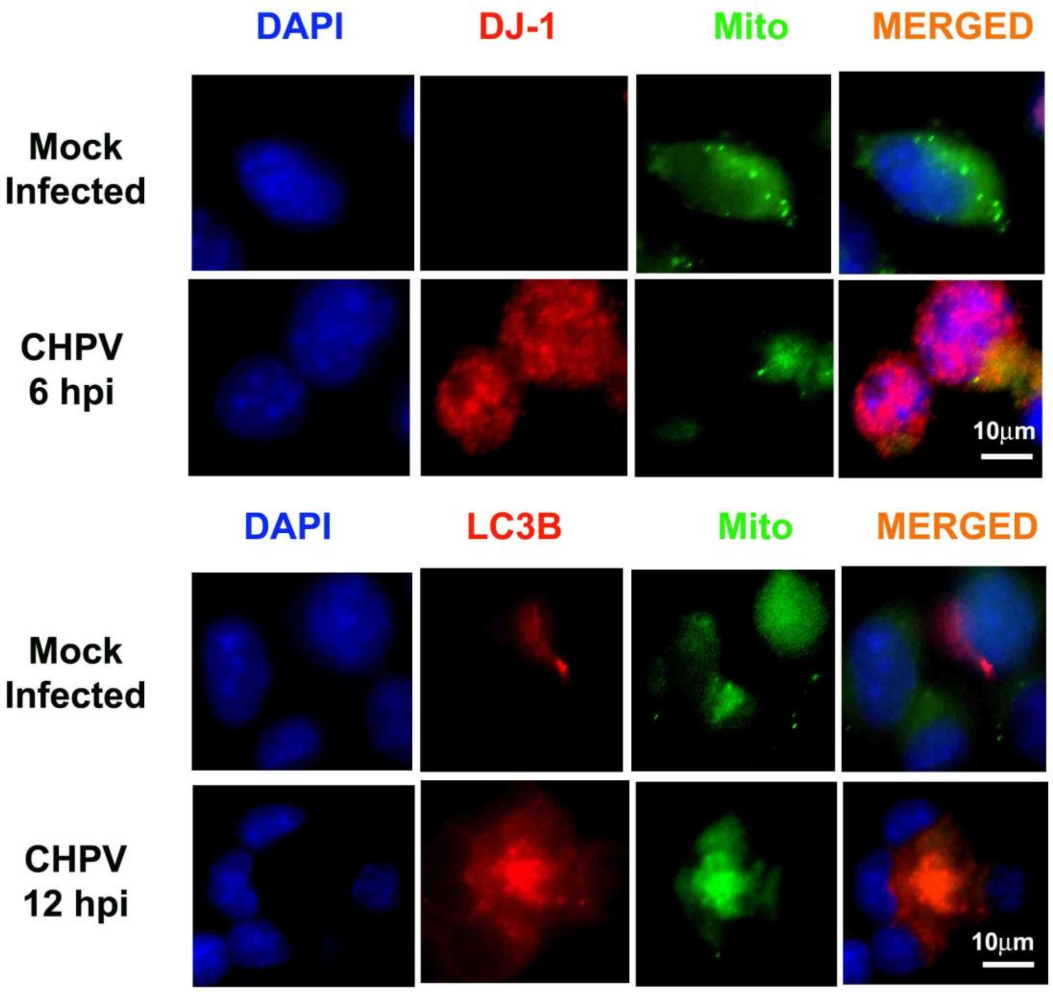

Fig. S2. Co-expression of Mito-Tracker (mitochondrial marker)/DJ-1 and Mito-Tracker/LC3B were analyzed using immunocytochemistry (ICC) from Neuro2A cells obtained from CHPV 6 hpi. Representative panel images demonstrate the results of ICC analysis. Scale= 10µm.

SREBP-2 or Sterol regulatory element-binding protein-2 are transcriptional activators of lipoproteins. Previous studies report that DJ-1 act as co-activators of various transcriptional factors including SREBP-2 that binds the promoter site of LDL receptor and initiate the transcription of LDL receptor (1) and regulates the cholesterol homeostasis (1). As reported previously cholesterol metabolism plays an important role in CHPV assembly in neurons (2). SREBP-2 activity is modulated by intracellular sterol level through a feedback inhibition mechanism via Insig-1 and -2 that bind to SREBP cleavage-activating protein (SCAP) in the ER and blocks the movement of the SCAP/SREBP complex to the Golgi complex. In response to a decrease in cholesterol level SCAP/SREBP complex disassociates to release SREBP that migrates to the Golgi where it gets proteolytically cleaved by protein convertase Subtilisin kexin isozyme/Site-1 protease (SKI-1/S1P) and the intramembranous metalloprotease Site-2 protease (S2P), that act sequentially to release the NH-terminal bHLH-Zip domain of SREBP-2 from the membrane. Dimerization of two N-terminal fragments facilitates binding to importin β. The entire complex hence enters the nucleus to bind to the sterol response element (SRE) in the promoter region of LDL receptor and up-regulate transcription (3, 4). The question arises as to how DJ-1 influences the migration of SREBP-2 into the nucleus? Previous reports say DJ-1 and other proteins having anti-oxidant activities lowers LDL cholesterol level (1, 5, 6). This lowered cholesterol level hence triggers the disassociation of SCAP/SREBP complex as described above.

Supporting the hypothesis LDL receptor expression significantly got reduced in DJ-1 knockdown cells while DJ-1 over-expression experiment showed increase in expression of LDL receptor (Fig. 6). We explored expression of DJ-1 and SREBP-2 from nuclear samples post CHPV infection at various time points. Expression of both the proteins increased at 12 hpi (Fig. S3 A & B). These findings were validated by DJ-1 over-expression samples.

The SRE binding site for LDL receptor promoter is a conserved sequence (7). In order to validate our study we obtained a mutated form of pLDLR-Luc SRE and introduced a point mutation to get a wild type form of the SRE binding site and measured the luciferase activity upon CHPV infection. With the infection of CHPV in neurons, it was expected to enhance the DJ-1 activity influencing the SREBP-2 migration into the nucleus and binding with the SRE site to promote LDL receptor expression. A luciferase tagged plasmid having a point mutation in a conserved SRE sequence of LDL receptor was obtained (pLDLR Mut-Luc SRE) from Addgene repository. The plasmid was subjected to point mutation at the same site to get the original sequence of pLDLR-LucSRE and investigated the binding of SREBP-2

post CHPV infection (Fig. S3 C).The luciferase assay result indicated a significant fold change in relative luciferase activity in case of the wild type when compared to the mutated form (p< 0.01). Hence it can be inferred from the above results that DJ-1 acts as co-activator of SREBP-2 in the nucleus to promote the transcription of LDL receptor post-CHPV infection. That further helped us to conclude that DJ-1 facilitates the CHPV assembly in neurons.

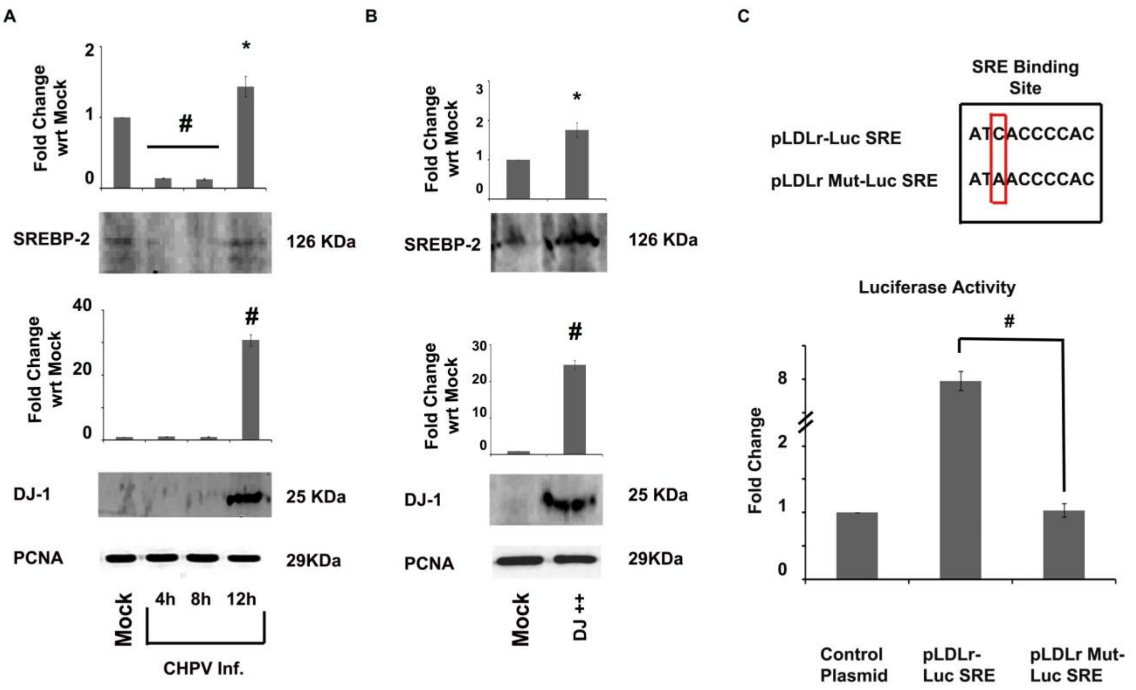

Figure S3: DJ-1 associates with SREBP-2 in nucleus and promotes LDL receptor transcription. Immunoblot analysis demonstrates the expression levels of SREBP-2, DJ-1 in nuclear proteins from a time-dependent study of CHPV infection (A) and DJ-1 over-expressing Neuro2A cells compared to their mock treated samples (B). PCNA was used as loading control in both the analyses (A & B). SRE binding site is a conserved sequence that is targeted specifically by SREBP proteins in order to promote the transcription of LDL receptors. A point mutation was introduced in the SRE binding site as shown in the figure in order to validate the binding of SREBP-2

to SRE binding site. The graph represents luciferase activity of LDL receptor Luc-SRE, LDL receptor Mut Luc-SRE and PGL2 plasmid CHPV 12 hpi. # represent $p < 0.01$. (n=3)

Tables:

Table S.1: **Identified Proteins**

**Protein Symbol**	**Protein Name**
DJ-1	Protein DJ-1/Park-7
HNRH1	Heterogeneous Nuclear Ribonucleoprotein H1 (H)
F161B	Family With Sequence Similarity 161, Member B
Polr2a	DNA-directed RNA polymerase II subunit RPB1
DPYSL3	(Dihydropyrimidinase-Like 3
PRDX6	Peroxiredoxin- 6
ITPA	Inosine Triphosphate Pyrophosphate
TUBB2B	Tubulin beta 2B
APOA1	Apolipoprotein A-I
LMNB1	Lamin-B1
ACTB	Actin B
VIME	Vimentin
YWHAG	14-3-3 protein gamma
DPYSL2	Dihydropyrimidinase-Like 2
Mpst	Mercaptopyruvate sulfurtransferase
COX5A	Cytochrome c oxidase
PEBP1	Phosphatidylethanolamine-Binding Protein 1
FABP7	Fatty Acid Binding Protein 7
ENO2	Enolase-2
HSPA5	Heat Shock 70kDa Protein 5
HEBP1	Heme-binding protein 1
Eif1ax	Eukaryotic translation initiation factor 1A
Ranbp1	Ran-specific binding protein 1

TBCA	Tubulin-specific chaperone A
Ppa1	Pyrophosphatase (Inorganic) 1
VCP	Valosin Containing Protein
STMN1	Stathmin-1
UQCRC1	Cytochrome b-c1 complex subunit 1
Cmpk1	Cytidine Monophosphate (UMP-CMP) Kinase 1
Pfn2	Profilin-2
GRB2	Growth factor receptor-bound protein 2
PGK2	Phosphoglycerate Kinase2
SOD1	Superoxide dismutase1
ERP29	Endoplasmic Reticulum Protein 29
Eif4h	Eukaryotic translation initiation factor 4H
TULP4	Tubby-related protein 4
HINT1	Histidine Triad Nucleotide Binding Protein 1
STIP1	stress-induced phosphoprotein 1
Rps12l1	ribosomal protein S12-like 1
ENO1	Enolase-1
IDH1	isocitrate dehydrogenase 1
TPI1	Triosephosphate Isomerase 1
BTRC	Beta-Transducin Repeat Containing E3 Ubiquitin Protein Ligase
CKB	Brain-type creatine kinase
PGAM1	phosphoglycerate mutase 1
PSMA2	Proteasome subunit alpha type-2
TAGL3	Transgelin-3
RBM3	RNA Binding Motif (RNP1, RRM) Protein 3
PPIA	Peptidylprolyl isomerase A
ALDOC	Aldolase C

Table S.2: **Differentially Expressed Proteins Identified from MALDI Analysis**

Spot Number	Protein Symbol	UniProt ID	*Protein Score	Matched Peptides	Mol. Wt. (Theo/Obs^K)	PI (Theo/ Obs^K)	Fold Change
1	DJ-1_MOUSE	Q99497	200	K.VTTHPLAK.D<br>K.DGLILTSR.GK.EI<br>LKEQESR.K<br>R.VEKDGLILTSR.G<br>K.VTVAGLAGKDP<br>VQCSR.D	20236/24000	6.38/6.8	2.06
2a	HNRH1_MOUSE	O35737	487	R.THYDPPR.K<br>R.VHIEIGPDGR.V	49454/76000	5.89/6.6	1.94
2b	F161B_MOUSE	Q8CB59	206	R.MASSPVSTAR.N	67269/76000	9.57/6.6	1.94
2c	Pol-2_MOUSE	P11369	33	R.TAMACAVR.S	150512/76000	9.71/6.6	1.94
3	DPYL3_MOUSE	Q62188	56	K.RIVAPPGGR.S<br>K.MADLHAVPR.G<br>K.SAADLISQAR.K	62296/80000	6.04/6.4	2.4
4	PRDX6_MOUSE	O08709	294	R.NFDEILR.V<br>R.VVFIFGPDK.K<br>K.LPFPIIDDK.G<br>K.LSILYPATTGR.N<br>R.DFTPVCTTELGR.<br>A<br>K.LKLSILYPATTGR.<br>N	24969/90000	5.72/5.8	2.5
5	ITPA_MOUSE	Q9D892	278	K.KIVFVTGNAK.K<br>R.GQTSGQIVMPR.<br>G<br>R.GQTSGQIVMPR.<br>G<br>K.LQEYFSVAAGAG<br>DH<br>K.IDLPEYQGEPDEI<br>SIQK.C	22225/30000	5.60/6.3	2.89
6	TBB2B_MOUSE	Q9CWF2	600	R.YLTVAAIFR.G<br>K.LAVNMVPFPR.L<br>R.FPGQLNADLRK.<br>L<br>R.AILVDLEPGTMD<br>SVR.S<br>K.GHYTEGAELVDS<br>VLDVVR.K	50377/54000	4.78/6.4	0.83

				MREIVHIQAGQCG<br>NQIGAK.F<br>K.FWEVISDEHGID<br>PTGSYHGDSDLQL<br>ER.I			
7	APOA1_MOUSE	Q00623	114	R.LSPVAEEFR.D<br>K.SNPTLNEYHTR.<br>A	30597/39000	5.31/5.6	1.92
8	LMNB1_MOUSE	P14733	158	R.FHQQGAPR.A<br>R.ASAPATPLSPTR.L	66973/110000	5.11/5.4	2.05
9	ACTB_MOUSE	P60710	185	K.AGFAGDDAPR.A<br>R.AVFPSIVGRPR.H<br>K.DSYVGDEAQSK<br>R.G<br>K.SYELPDGQVITIG<br>NER.F	42052/58000	5.29/5.4	0.59
10	VIME_MOUSE	P20152	321	R.LGDLYEEEMR.E<br>K.NLQEAEEWYK.S<br>R.SLYSSSPGGAYV<br>TR.S<br>R.MFGGSGTSSRPS<br>SNR.S<br>R.DGQVINETSQHH<br>DDLE.	53712/74000	5.05/5.2	0.6
11	1433G_MOUSE	P61982	131	R.LAEQAER.Y<br>R.YDDMAAAMK.N<br>K.NVTELNEPLSNE<br>ER.N	28456/40000	4.80/5.0	3.0
12	DPYL2_MOUSE	O08553	58	R.IVAPPGGR.A<br>K.SAAEVIAQAR.K	62638/20000	5.95/6.2	6.7
13	THTM_MOUSE	Q99J99	203	R.RFQVVDAR.A<br>R.FQGTQPEPR.D<br>K.LLDASWYLPK.L<br>R.AQPEHIISEGR.G<br>R.HIPGAAFFDIDR.<br>C<br>R.AAGRFQGTQPEP<br>R.D	33231/42000	6.12/6.1	0.6
14	COX5A_MOUSE	P12787	117	R.RLNDFASAVR.I<br>K.GMNTLVGYDLV<br>PEPK.I<br>K.GMNTLVGYDLV<br>PEPK.I	16319/16000	5.01/5.0	1.8

15	PEBP1_MOUSE	P70296	180	K.FKVETFR.K<br>K.LYTLVLTDPDAPSR.K	20988/23000	5.19/5.0	1.99
16	FABP7_MOUSE	P51880	288	K.ALGVGFATR.Q<br>R.LDGDKLIHVQK.W<br>K.LTDSQNFDEYMK.A<br>K.LTDSQNFDEYMK.A<br>K.MVVTLTFGDIVAVR.C<br>K.MVVTLTFGDIVAV<br>R.QVGNVTKPTVIISQEGGKVVIR.T<br>K.NTEINFQLGEEFEETSIDDR.N<br>K.NTEINFQLGEEFEETSIDDRNCK.S	15169/17000	5.47/5.2	1.87
17	ENOG_MOUSE	P17183	561	R.DLPLYR.H<br>R.LGAEVYHTLK.G<br>R.IEEELGDEAR.F<br>K.AGAAERDLPLYR.H<br>R.FAGHNFRNPSVL.1<br>R.GNPTVEVDLYTAK.G<br>K.MVIGMDVAASEFYR.D<br>K.MVIGMDVAASEFYR.D<br>R.AAVPSGASTGIYEALELR.D<br>R.YITGDQLGALYQDFVR.N<br>K.LAMQEFMILPVGAESFR.D<br>K.LAMQEFMILPVGAESFR.D	47609/75000	4.99/5.1	1.61
18	GRP78_MOUSE	P20029	560	K.DAGTIAGLNVMR.I	72492/150000	5.01/5.0	5.4

				R.TWNDPSVQQDIK.F K.SDIDEIVLVGGSTR.I R.AKFEELNMDLFR.S			
19	HEBP1_MOUSE	Q9R257	386	K.EADYVAHATQLR.T R.EGITVYSTQFGGYAK.E K.FATVEVTDKPVDEALR.E R.IPNQFQGSPPAPSDESVK.I R.IPNQFQGSPPAPSDESVKIEER.E	21167/30000	5.18/5.2	1.88
20	IF1AX_MOUSE	Q8BMJ3	250	K.AYGELPEHAK.I R.LEAMCFDGVKR.L R.SLKAYGELPEHAK.I K.VWINTSDIILVGLR.D K.KVWINTSDIILVGLR.D R.ELVFKEDGQEYAQVIK.M	16564/28000	5.07/5.1	2.07
21	RANG_MOUSE	P34022	299	R.LEELQR.E K.DNTVHDLR.Q K.DKFMLQAK.V R.LMAKVEDMQR.N K.NLNNCLQQLK.Q R.LMAKVEDMQR.N R.NSQGTSAEGSVRK.E R.EHNSILETALAK.R	23753/46000	5.15/5.1	2.13

				K.HKAYENAVSILS R.R R.NILSKDNTVHDL R.Q R.LEEFEGEREQLQ K.V .MDGASAKQDGLW ESK.S K.LQASQAEITSLQ HAR.Q K.SNQVEHLQQETA TLR.K K.SELEMVQEDLSE TQK.D K.SELEMVQEDLSE TQK.D R.LPDQQDTAQDAS VEVNR.G R.ALEVELQNVGQS KILLEK.E R.LQGEMAHIQVG QMTQAGLLEHLK. L			
22	TBCA_MOUSE	P48428	63	R.MMIPDCQR.R K.QAEILQESR.M	12807/18000	5.25/5.3	2.69
23	IPYR_MOUSE	Q9D819	112	R.AAPFTLEYR.V K.GISCMNTTVSES PFK.C	33102/55000	5.37/5.4	0.51
24	TERA_MOUSE	Q01853	195	R.GILLYGPPGTGK. T K.GVLFYGPPGCGK .T R.WALSQSNPSALR. E K.AIANECQANFISI K.G	89950/190000	5.14/5.4	8.8
25	TERA_MOUSE	Q01853	164	R.GILLYGPPGTGK. T K.GVLFYGPPGCGK .T R.WALSQSNPSALR. E K.AIANECQANFISI	89950/19000	5.14/5.6	1.8

				K.G			
26	STMN1_MOUSE	P54227	60	K.RASGQAFELILSPR.S	17264/18000	5.76/5.7	2.45
27	TERA_MOUSE	Q01853	169	R.DHFEEAMR.F R.GILLYGPPGTGK.T K.GVLFYGPPGCGK.T R.WALSQSNPSALR.E	89950/180000	5.14/5.7	4.92
28	QCR1_MOUSE	Q9CZ13	103	R.VYEEDAVPGLTPCR.F	53446/50000	5.34/5.8	4.69
29	QCR1_MOUSE	Q9CZ13	101	R.VYEEDAVPGLTPCR.F	53446/18000	5.34/6.0	4.73
30	KCY_MOUSE	Q9DBP5	412	K.NKFLIDGFPR.N R.NQDNLQGWNK.T K.YGYTHLSAGELLR.D K.IVPVEITISLLKR.E R.KNPDSQYGELIEK.Y K.YGYTHLSAGELLRDER.K	22379/30000	5.68/6.0	2.44
31	PROF2_MOUSE	Q9JJV2	253	K.SQGGEPTYNVAVGR.A R.DSLYVDGDCTMDIR.T R.TKSQGGEPTYNVAVGR.A R.DSLYVDGDCTMDIR.T	15364/15000	6.78/6.3	3.60
32	GRB2_MOUSE	Q60631	238	R.HDGAFLIR.E R.NYVTPVNR.N K.FGNDVQHFK.V K.QRHDGAFLIR.E K.GACHGQTGMFPR.N K.GACHGQTGMFPR.N	25336/2000	5.89/5.3	3.06

				R.ESESAPGDFSLSVK.F K.FNSLNELVDYHR.S			
33	PGK2_MOUSE	P09041	60	K.INAQIVAQAK.L	45223/15000	6.36/6.2	2.63
34	SODC_MOUSE	P08228	141	K.KHGGPADEER.H R.VISLSGEHSIIGR.T	16104/18000	6.03/6.5	4.0
35	ERP29_MOUSE	P57759	185	K.ESYPVFYLFR.D K.ILDQGEDFPASEMAR.I K.ILDQGEDFPASEMAR.I	28862/47000	5.74/6.7	2.42
36	IF4H_MOUSE	Q9WUK2	179	R.FRDGPPLR.G R.SLRVDIAEGR.K R.GSNMDFREPTEEER.A	27381/47000	6.91/6.9	2.05
37	TULP4_MOUSE	Q9JIL5	66	R.GHNSEVVLVR.W R.TAQPTVPNPLK.L	171128/55000	7.30/7.0	4.67
38	HINT1_MOUSE	P70349	123	K.IIFEDDR.C K.AQVAQPGGDTIFGK.I	13882/15000	6.39/6.8	3.11
39	STIP1_MOUSE	Q60864	440	K.HYTEAIKR.N K.LMDVGLIAIR. K.LMDVGLIAIR. K.TVDLKPDWGK.G R.IGNSYFKEEK.Y K.YKDAIHFYNK.S K.LDPQNHVLYSNR.S R.LAYINPDLALEEK.N K.ALDLDSSCKEAADGYQR.C	63170/101000	6.40/7.2	2.26
40	RS12_MOUSE	P63323	150	K.DVIEEYFK.C K.LGEWVGLCK.I K.TALIHDGLAR.G K.ESQAKDVIEEYFK.C	14858/15000	7.02/7.1	2.67
41	ENOA_MOUSE	P17182	119	R.IGAEVYHNLK.N	47453/69000	6.36/7.3	0.44

42	IDHC_MOUSE	O88844	200	K.GWPLYLSTK.N<br>K.SEGGFIWACK.N<br>R.LVTGWVKPIIIGR.H<br>R.AKLDNNTELSFFAK.A<br>K.GQETSTNPIASIFAWSR.G	47044/69000	6.72/7.3	2.01
43	TPIS_MOUSE	P17751	548	K.IAVAAQNCYK.V<br>K.SNVNDGVAQSTR.I<br>K.LDEREAGITEK.V<br>K.VIADNVKDWSK.V<br>R.IIYGGSVTGATCK.E<br>K.TATPQQAQEVHEK.L<br>K.DLGATWVVLGHSER.R<br>K.VVLAYEPVWAIGTGK.T<br>R.RHVFGESDELIGQK.V<br>K.TATPQQAQEVHEKLR.G	32684/42000	5.56/7.4	0.34
44	FBW1A_MOUSE	Q3ULA2	56	R.FNNGMMVTCSK.D<br>R.SIAVWDMASPTDITLR.R	70304/77000	8.19/7.6	0.44
45	KCRB_MOUSE	Q04447	541	R.DWPDAR.G<br>K.LLIEMEQR.L<br>K.GGNMKEVFTR.F<br>K.GGNMKEVFTR.F<br>R.GFCLPPHCSR.G<br>K.VLTPELYAELR.A<br>R.FCTGLTQIETLFK.S<br>K.LAVEALSSLDGDLSGR.Y<br>R.GTGGVDTAAVGGVFDVSNADR.L<br>K.LAVEALSSLDGD	42971/70000	5.4/6.7	0.10

				LSGRYYALK.S			
46	TERA_MOUSE	Q01853	187	R.GILLYGPPGTGK.T K.GVLFYGPPGCGK.T R.WALSQSNPSALR.E K.AIANECQANFISIK.G	89950/150000	5.14/5.2	0.10
47	PGAM1_MOUSE	Q9DBJ1	507	R.HYGGLTGLNK.A R.VLIAAHGNSLR.G R.HGESAWNLENR.F R.HYGGLTGLNKAETAAK.H R.ALPFWNEEIVPQIK.E	28928/37000	6.75/7.2	0.09
48	PSA2_MOUSE	P49722	272	K.SILYDER.S R.LTPTEVRDYLAAIA K.HIGLVYSGMGPDYR.V K.HIGLVYSGMGPDYR.V	26024/35000	7.12/7.3	0.14
49	TAGL3_MOUSE	Q9R1Q8	298	R.GEPSWFHR.K R.RGFSEEQLR.Q K.GASQAGMTGYGMPR.Q K.LINSLYPPGQEPIPK.I	22627/22000	6.84/7.1	0.09
50	RBM3_MOUSE	O89086	250	R.QIRVDHAGK.S R.DYSGSQGGYDR.Y R.YSGGNYRDNYDN R.SRDYSGSQGGYDR.Y R.YDSRPGGYGYGYGR.S R.GFGFITFTNPEHASDAMR.A R.GFGFITFTNPEHA	16595/17000	6.84/6.9	0.08

				SDAMR.A			
51	PPIA_MOUSE	P17742	104	R.VSFELFADKVPK.T K.VKEGMNIVEAMER.F R.IIPGFMCQGGDFTR.H	18131/19000	7.73/7.6	0.15
52	ALDOC_MOUSE	P05063	476	K.ELSDIALR.I R.ALQASALNAWR.G R.DNAGAATEEFIKR.A R.LSQIGVENTEENRR.L R.YEGSGDGGAAAQSLYIANHAY.- K.ISDRTPSALAILENANVLAR.Y K.VDKGVVPLAGTDGETTTQGLDGLLER.C R.TVPPAVPGVTFLSGGQSEEEASLNLNAINR.C	39769/51000	6.79/7.3	0.09
53	ALDOC_MOUSE	P05063	519	K.ELSDIALR.I R.ALQASALNAWR.G R.DNAGAATEEFIKR.A R.LSQIGVENTEENRR.L R.YEGSGDGGAAAQSLYIANHAY K.ISDRTPSALAILENANVLAR.Y K.VDKGVVPLAGTDGETTTQGLDGLLER.C R.TVPPAVPGVTFLSGGQSEEEASLNLNAINR.C	39769/60000	6.79/7.2	0.15

***Protein Score:** Protein score is -10*Log(P), where P is the probability that the observed match is a random event. Protein scores greater than 55 are significant ($p<0.05$). Protein scores are derived from ions scores as a non-probabilistic basis for ranking protein hits.

Mol. Wt: Molecular Weight

pI: Isoelectric Point

κ: Approximate Value

Fold Change: Fold change with respect to control.

References:

1. **Yamaguchi S, Yamane T, Takahashi-Niki K, Kato I, Niki T, Goldberg MS, Shen J, Ishimoto K, Doi T, Iguchi-Ariga SM, Ariga H.** 2012. Transcriptional activation of low-density lipoprotein receptor gene by DJ-1 and effect of DJ-1 on cholesterol homeostasis. PLoS One **7:**e38144.
2. **Ghosh S, Mukherjee S, Basu A.** 2015. Chandipura virus perturbs cholesterol homeostasis leading to neuronal apoptosis. J Neurochem **135:**368-380.
3. **Ye Q, Lei H, Fan Z, Zheng W, Zheng S.** 2014. Difference in LDL receptor feedback regulation in macrophages and vascular smooth muscle cells: foam cell transformation under inflammatory stress. Inflammation **37:**555-565.
4. **McFarlane MR, Liang G, Engelking LJ.** 2014. Insig proteins mediate feedback inhibition of cholesterol synthesis in the intestine. J Biol Chem **289:**2148-2156.
5. **Khor HT, Ng TT.** 2000. Effects of administration of alpha-tocopherol and tocotrienols on serum lipids and liver HMG CoA reductase activity. Int J Food Sci Nutr **51 Suppl:**S3-11.
6. **Hirata F, Fujita K, Ishikura Y, Hosoda K, Ishikawa T, Nakamura H.** 1996. Hypocholesterolemic effect of sesame lignan in humans. Atherosclerosis **122:**135-136.
7. **Seo YK, Chong HK, Infante AM, Im SS, Xie X, Osborne TF.** 2009. Genome-wide analysis of SREBP-1 binding in mouse liver chromatin reveals a preference for promoter proximal binding to a new motif. Proc Natl Acad Sci U S A **106:**13765-13769.
